# Interactive Effects of Heavy Metals and Co-Occurring Abiotic Stresses in Plants: Shared Response Mechanisms and Mitigation Approaches

**DOI:** 10.3390/plants15142245

**Published:** 2026-07-22

**Authors:** Maurizio Capuana, Emily Rose Palm

**Affiliations:** 1 IBBR–Institute of Biosciences and Bioresources, National Research Council (CNR), 50019 Firenze, Italy; 2 Department of Biotechnology and Biosciences, University of Milano-Bicocca, 20126 Milano, Italy

**Keywords:** drought, flooding, heat, hydrocarbons, oxidative stress, pesticides, plastics, salinity, stress mitigation, tolerance

## Abstract

Heavy metal (HM) contamination of soils frequently occurs in combination with other environmental stressors, resulting in a cumulative effect that interferes with plant physiology, biochemistry, and development in a more severe and complex manner than the individual stressors alone. While there are many reviews that consider abiotic stress combinations broadly, this review focuses specifically on combined HM + abiotic stress conditions and classifies in terms of the effect on symptoms or induced defense mechanisms as additive, synergistic (exacerbated) or even antagonistic (counteracted). It is revealed that the combined stress response may depend on the way that each of the stresses presents itself to plant tissues, i.e., whether it is a chemical compound whose accumulation into roots may be regulated or a stress whose application is more general, such as heat stress or flooding (localized versus systemic, respectively). Combined localized stresses such as HMs and hydrocarbons tend to elicit synergistic responses, while localized + systemic stresses are more variable, tending toward antagonistic, such as HM and drought. Modern analysis tools indicate an overlap in or induction of unique response pathways that may alter depending on the conditions (intensity, duration and timing of exposure; greenhouse versus field conditions). The latest findings in mitigation techniques, ranging from chemical amendments and biological treatments, such as biochar and phytohormones, respectively, to genetic engineering, as also summarized. It is revealed that biochar and genetic approaches are both the most-widely used and the most effective.

## 1. Introduction

**Figure 1:**
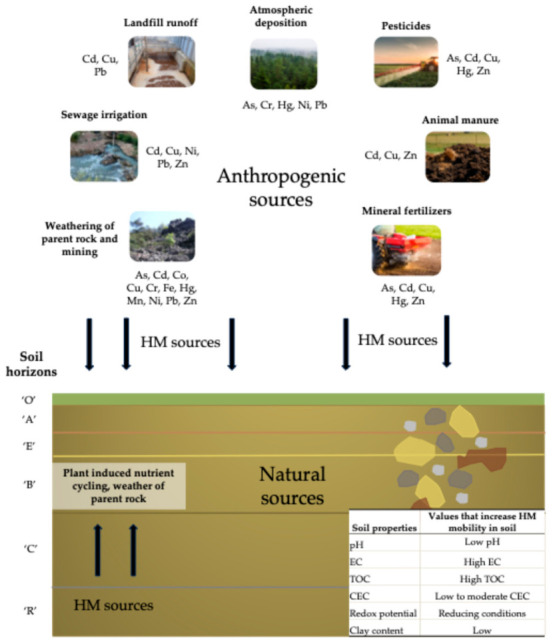
A summary of HM sources and factors regulating HM mobility in the soil and availability to plants. Large black arrows indicated the direction of flow from HM sources. Soil horizons: O—organic matter; A—topsoil; E—eluviation layer; B—subsoil; C—parent rock; R—bedrock.

Plants must cope with a wide range of abiotic stresses that impact their ability to obtain resources from the environment. Among them, heavy metals (HM) pollution is an ever-increasing environmental problem with a strong negative influence on plant life and agricultural production [1]. Agricultural soils themselves may present high levels of HM and metalloids, such as cadmium (Cd), chromium (Cr), copper (Cu), manganese (Mn), lead (Pb), nickel (Ni), zinc (Zn), arsenic (As) and mercury (Hg). Recent estimates suggest that between 14 and 17% of agricultural soils are contaminated with at least one heavy metal (Table 1) [2,3,4,5,6,7,8,9], translating to upwards of 8 million hectares of arable land affected by heavy metals [2]. In this same study, a machine learning model approach (extremely randomized trees) was used to aggregate soil data from over 700,000 samples representing 1493 different climatic, geographic and land use regions. It emerged that both natural (geological and climatic) and anthropogenic factors have contributed to the formation of a metal enriched belt that stretches across southern Europe, the Middle East, southern India and southeast China. This area has the highest probability of soils exceeding the threshold limits for agricultural areas for seven metals: As, Cd, Co, Cr, Cu, Ni and Pb. Many sources of HM contamination have been identified (Figure 1) [10,11,12,13]. Among these, the main ones include certain industrial emissions, such as those from mining, smelting, and manufacturing; in agriculture, the use of phosphate fertilizers containing Cd and pesticides containing As compounds; in urban areas, the predominant sources of HMs are electronic waste and untreated wastewater. These various toxic substances can thus easily infiltrate food supply chains and consequently threaten the safety of products intended for human and animal consumption [14,15].

The increasing presence of HM in soils (and waters) has become a critical priority not simply for sustainable global development, but in particular for food security and human health. HM have a very long half-life, and therefore persist in the environment for extended periods, thus exerting toxic effects on microorganisms, plants, animals, and humans [16]. Highly toxic metal species can bioaccumulate within the food chain, intensifying the risk to human health. Specific HM and metalloids, in fact, may alter human metabolic processes through chronic low-level exposure [17,18]. Several substances, such as Hg, Cd, Pb, and inorganic As species, may cause cytotoxicity and systemic toxicity. For instance, they can bind to enzymes in mammalian cells, interact with key cellular components, induce disruption of redox homeostasis and ion transport mechanisms, and compromise DNA repair processes [17,18,19,20].

Unfortunately, HMs represent only a portion of the environmental stressors that threaten the growth, productivity and survival of plants. Climate change is aggravating the frequency and intensity of abiotic stresses such as drought, high temperatures and flooding [21]. Anthropogenic factors such as the pressure to stimulate greater crop productivity and the increased production of waste is resulting in greater inputs of chemicals and microplastics into agricultural soils and irrigation water. Drought (moderate) and heat stress currently affect the greatest amount of agricultural land area (Table 1). A recent study estimated the global crop losses by the end of the century due to climate change for six staple crops under current emission scenarios as −27.8% for maize, −6% for rice, −21.7% for sorghum, −35.7% for soybean, −28% for wheat and −29.8% for cassava [22]. These estimates also took into account different adaptation strategies (e.g., locally adapted cultivars, water management strategies), finding that countries with moderate temperature and precipitation regimes are the most vulnerable to crop losses because their production systems are inherently less adapted to higher temperatures and drought. This suggests that the losses will not be distributed evenly across the globe and that adapting our production methods will not be sufficient.

Alarmingly, climate change is also increasing the probability of HM toxicity co-occurring with other abiotic stresses and increasing the pressure to find targeted mitigation strategies to reduce the impact to our agricultural production system. Climatic factors such as increased temperature and intense precipitation events, as well as elevated transpiration rates of plants, are increasing the availability and mobility of heavy metals in soil [5]. Until recently, research has focused primarily on individual stress responses in plants, as well as single stress-specific mitigation methods. However, rapidly changing environmental conditions and future climate models are stimulating a change in experimental approach to look further into combined stress responses in plants. These phenomena have been recently analyzed and reviewed in crop plants with multiomic approaches revealing the fact that differential regulation occurs depending on whether plants experience stresses individually or in combination based on complex stress response networks [23,24,25]. These broad reviews have been fundamental in identifying physiological and molecular processes that are involved in plant responses to combined abiotic stress. Primary and secondary metabolism are among the key processes most commonly impacted [23]. Both are targeted by the elevated production of reactive oxygen species (ROS) that is a general physiological response to HMs and many other abiotic stresses including drought, salinity and high temperature [1]. These processes are intricately connected to tissue chlorosis, reductions in shoot and root growth and the downregulation of photosynthesis. The tendency of current reviews to consider heavy metals as single group ends up generalizing their effects on plant physiology and comparing them as a monolith in relation to other abiotic stress combinations. However, heavy metals may be present in diverse ionic forms which can influence their availability for uptake, their transport into plant tissues and their toxicity thresholds. Some heavy metals are micronutrients and are therefore essential to the plant life cycle in small quantities, while others have no role in plant function. By focusing attention on individual HMs in combination with other abiotic stresses, this review seeks to elucidate response patterns that may be connected to specific mechanisms involved in the uptake, accumulation and tolerance to HMs. This information should shed light on the mitigation strategies to be applied that are most adapted to specific case scenarios.

Given the vulnerability of our global food production system to variability in soil and weather conditions, and the increasing likelihood of HM and abiotic stress co-occurrence, going forward it will be vital to know which interactions have the greatest negative impact on plant health and why. This is especially important in the areas where the highest rate of HM contamination is occurring in coincidence with zones of elevated agricultural productivity [2]. The present review focuses on the seven HMs with the highest probability of affecting agricultural soils at a global scale (As, Cd, Co, Cr, Cu, Ni and Pb) in combination with other common abiotic stresses—salinity, drought, flooding, temperature, industrial chemicals and microplastics. Following an overview of transport mechanisms for the selected HMs mentioned above and general abiotic stress responses in plants, connections between physiological processes and response mechanisms will be identified in order to classify combined HM + abiotic stress responses as additive, synergistic and antagonistic—and the likely mechanisms involved (e.g., biochemical mitigation, physical exclusion). The emerging mitigation strategies that can aid in shifting the response to one that is antagonistic are also presented, including biological, chemical and genetic approaches. These studies reveal the advances in knowledge that have been made regarding the complex HM and combined abiotic stress responses and suggest new areas of research to improve the crop adaptation to future conditions.

## 2. Literature Review Approach

In contrast to other reviews in the literature that tend to consider HMs as a single group, this review focuses on individual HMs in combination with other abiotic stresses. In particular, studies dealing with combinations of abiotic stress and the seven HMs with the highest probability to increase in concentration in agricultural soils (As, Cd, Co, Cr, Cu, Ni and Pb) were included in this review. These were limited to studies on agriculturally relevant crop species, with some exceptions for plants with potential commercial value in the future (current local use, halophytes). The principal search engines used were PubMed, Google Scholar and Researchgate. Studies were selected based on the time since publication (2006–present for physiological studies and 1996–present for more general information on plant responses), plant species (crop species, mainly herbaceous) and type of study conducted (controlled greenhouse and growth chamber trials and pot experiments). Table 2 summarizes the specific keyword and themes used in the search process, and a list of criteria used to determine inclusion or exclusion of studies from consideration. In particular, studies on plants with no edible or commercial production as food, building materials or fibers were not included, and neither were those focused solely on metals outside of the seven listed above (ex. Al, Hg, Sr, Ti, Zn). Though relevant, these excluded metals are less likely to have an outsized effect on global food production in the future based on current predictions. However, studies with multiple HMs that included at least one metal from the specified list were considered. The literature search focused on the physiological responses of plants to the combination of individual abiotic stresses with HM stresses, introduced by notes on the action of each stress source. We looked especially for studies that used multiomic approaches to characterize changes in gene expression and metabolic pathways in response to stress intensity. Also, of particular interest were trials that explicitly described the type of response elicited with terms such as additive, synergistic and antagonistic, as these types of responses can shift depending on the intensity and combination of the stress applied.

## 3. Heavy Metal Sources, Mobility and Effects on Plants

Heavy metals in soil originate from both biogeochemical and anthropogenic sources (Figure 1). The weathering of soil parent material is a natural source of HMs that can depend on both climatic and geographic factors, especially with regard to As, Cd, Co, and Cu [2]. Anthropogenic activities are rapidly increasing the amount of HMs deposited in agricultural soils through multiple methods of diffusion. Run-off from automobiles, landfill leachates, mining activities and industrial operations significantly contribute to soil HM concentrations. The agricultural industry itself is also responsible for increasing soil HM levels, especially through the application of pesticides (in particular As and Pb) and through irrigation with contaminated water [11,12]. The abovementioned waste management, mining and industrial activities all risk releasing HMs into underground aquifers that are then used for crop irrigation. As such, the principal anthropogenic sources of HMs are highly dependent on the types of activities that exist in a region and whether one is looking at the agricultural system of a region as a whole or at the scale of individual fields. For example, in England, it was found that atmospheric deposition was responsible for up to 82% of HMs in the soil; however, when evaluating at the level of individual fields, sewage was a significant factor in the cases where it was applied [11]. Similarly, in a meta-analysis of HM sources in agricultural soils in China, atmospheric deposition caused between 43 and 82% of the As, Cr, Hg, Ni and Pb contents of soil, while significant concentrations of Cd, Cu and Zn came from livestock manure applications [12]. Livestock manure is beginning to receive greater attention as it represents the circularity of HMs in the food chain: animals are fed with HM- and growth promoter-enriched feed and a portion of the HMs consumed ends up in manure [13,26]. The manure is then applied to agricultural fields as fertilizer, with the additional consequence of increasing soil HM concentrations.

The mobility of HMs and their availability to plants is mainly determined by soil properties (Figure 1). Soil pH, electroconductivity (EC), and total organic content (TOC) are the three factors that have the greatest impact on HM mobility and availability [27,28,29,30]. pH is negatively correlated with HM availability, as demonstrated in rice plants in which root HM concentrations of Cr, Cu, Fe, Mn and Pb increased with soil pH values below 7.0, but decreased when pH rose above 7.0 [30]. In contrast, EC and TOC are positively correlated with HM availability. In particular, TOC together with soil clay content, affect the cation exchange capacity of soil by providing negatively charged particles to which HMs and other essential nutrients can bind or be released from into the soil solution for uptake by plants. Redox potential of soil can affect HM mobility with greater HM mobilization under reducing conditions, as found with As, Fe and Mn [31]. Additionally, plant–soil cycling helps to mobilize HMs from lower soil horizons to upper horizons within the range of plant roots [32]. Clearly, soil chemistry and texture strongly influence the degree to which HMs are available for uptake by plants once they are deposited into the soil by the abovementioned sources.

**Figure 2:**
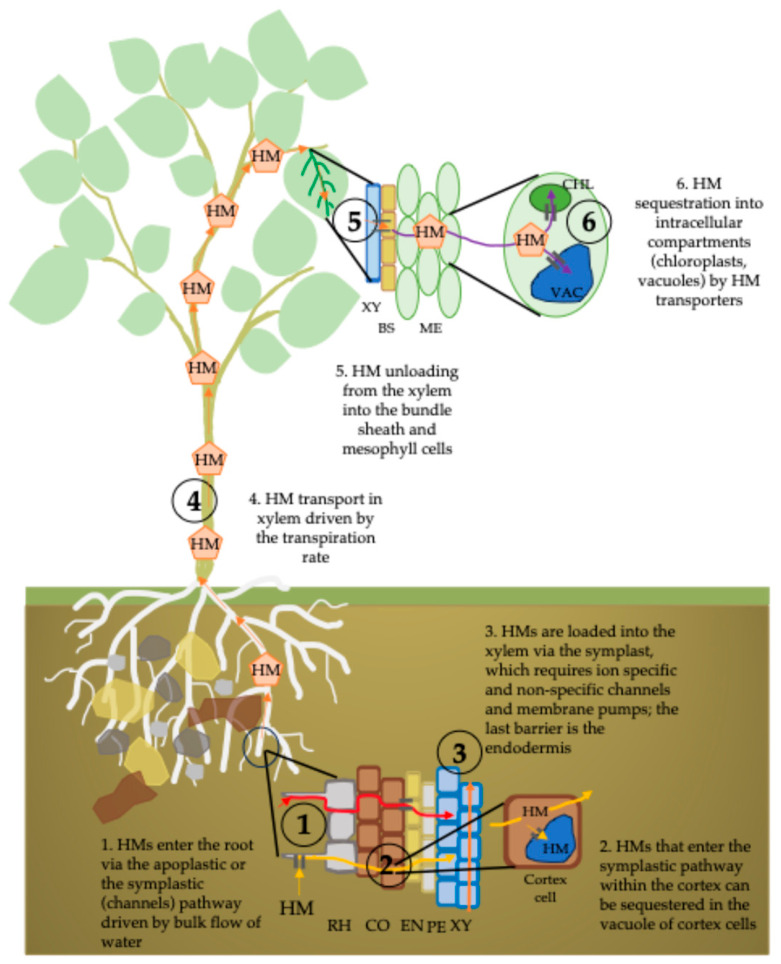
HM transport from soil to the leaf via xylem uploading and unloading. Cell types: RH—root hair; CO—cortex; EN—endodermis; PE—pericycle; XY—xylem; BS—bundle sheath; ME—mesophyll; VAC—vacuole; CHL—chloroplast. Red arrow: apoplastic pathway; yellow arrow: symplastic pathway; dark orange arrow: vascular transport; purple arrow: intercellular transport (leaf).

HMs enter plants through absorption by the root system and may remain there or be translocated to the shoots, reaching toxic concentrations as they accumulate (Figure 2) [33]. In the absence of exclusion or avoidance mechanisms, such as chelation by root exudates, heavy metal ions such as Cd^2+^ and Pb^2+^ and Zn^2+^ in the soil may take advantage of non-selective cation channels to enter plant roots where they may accumulate or be translocated to the shoots, depending on the species [33,34,35]. This process is highly regulated by the presence of numerous transporters, both specific and non-specific for HMs. Long-distance transport of HMs, especially translocation from the roots to the shoots, occurs via the transpiration stream in the xylem tissue. Transporter proteins at the root endodermis and bundle sheath cells of leaves determine HM loading and unloading of the xylem. In both root cortical cells and leaf parenchyma cells, HMs can accumulate in organelles at the subcellular level or be sequestered in vacuoles. Thus, the specificity, localization and abundance of transporters determine the distribution of HMs within plant tissues. This reliance on membrane-bound transporters is a key factor that may explain similarities between salinity and HM stress responses (glycophytes versus halophytes; metallophytes) and differentiate HM stress from other abiotic stresses such as temperature or drought. The various transporters and their specificities also help to distinguish the threshold levels of different plant species for the various HMs.

**Figure 3:**
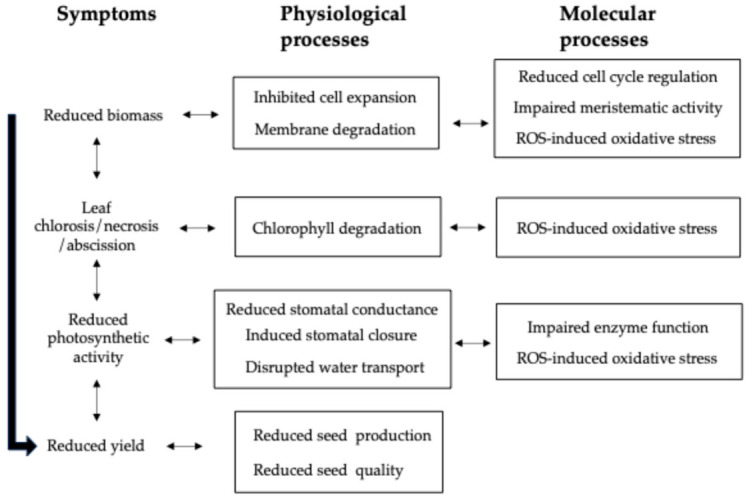
A summary of the main effects of heavy metal stress on plants, linking symptoms with the underlying physiological and molecular processes.

The distribution of accumulated HMs within plant tissues also determines the degree and location where HM stress responses and symptoms may be stimulated. Figure 3 summarizes the common HM physiological responses seen at three different organ levels in plants: roots, shoots and leaves. The negative effects of toxic concentrations of heavy metals range from morphological alterations in plant growth to disruptions in fundamental physiological processes such as photosynthesis and cellular respiration. Increased ROS production plays a pivotal role in determining the extent and type of damage that occurs, including cessation of cell proliferation at meristems and the degradation of membranes. Prolonged exposure can lead to stress effects both from the direct impacts of the HMs themselves as well as the limitations in nutrient and water acquisition due to reductions in root biomass.

There are many overlapping effects between the different heavy metals, indicating that there are shared response patterns involving fundamental physiological processes and/or mechanisms of stress mitigation [25,36]. An increase in ROS production is one of the first markers of HM stress, leading to the induction of both enzymatic and nonenzymatic antioxidant systems and oxidative stress responses with prolonged or intense exposure [36]. The visual and physiological effects that are summarized in Figure 3 are regulated following the activation of several signaling networks in response to HMs [37,38,39,40,41,42,43,44,45]. Stress response gene expression is regulated by a number of signaling cascade networks including Ca-binding kinases (CKPKs, CMLs, CaM [42]), MAPKs [43], enzymatic and non- enzymatic antioxidants [45] and hormones like abscisic acid (ABA), ethylene [46] and jasmonic acid (JA) [47]. Thus, different heavy metals may activate similar biochemical and genetic pathways to induce a catalog of plant responses and defense mechanisms.

## 4. Effects of Other Individual Abiotic Stresses

The way that an abiotic stress factor initially interacts with a plant and whether the effects of that stress are localized or systemic are likely two key factors that may aid our understanding of the responses and defense mechanisms that are stimulated in the plant following exposure. This section briefly looks at individual abiotic stresses other than HMs in terms of their interaction or mode of perception in plants and classifies them as localized or systemic stresses (Table 3; [48,49,50,51,52,53,54,55,56,57,58,59,60,61,62,63,64,65]). Localized stress in this instance is defined as a stress factor whose impact on plant tissues is mainly determined by its distribution, i.e., where it is accumulated. These are compounds whose movement within the plant body is regulated by transporters, as seen with HMs, hydrocarbons, MPs and other synthetic chemicals. In contrast, systemic stress factors are those that are applied ubiquitously across the plant body and stimulate stress responses factors that are transported long-distance such as ROS and plant hormones to initiate a global response. These include drought, flooding and temperature stress, which tend to affect the entire below- or aboveground organ system, and sometimes both, simultaneously. Here, salinity stress is listed as both localized and systemic because though sodium (Na) ion accumulation and distribution are regulated by transporters, it often stimulates drought symptoms by disturbing plant water relations, notably osmolarity, and thus water potential.

### 4.1. Localized and Mechanical Stresses: Microplastics, Hydrocarbons, Synthetic Chemicals

Agricultural productivity has long been associated with the use of a broad spectrum of synthetic chemical products including herbicides, pesticides and fungicides. These substances, together with heavy metals, are among the most dangerous contaminants of agricultural soils, causing major environmental problems globally [60]. Recent studies have also shown that the amount of microplastics (MPs) in aquatic and land ecosystems is dramatically increasing, with effects that can be transferred between organisms through food webs. Chemical compounds like pesticides, hydrocarbons and microplastics can be accumulated by plants to toxic levels that damage their tissues in ways similar to HMs. Also, like HMs, their uptake and transport can be regulated, depending on the plant species and the chemical itself, limiting the effects to the tissue with direct exposure. Hydrocarbons, such as perfluoroakyl and polyfluoroakyl substances (PFAs) are commonly used in industrial processes and are frequent environmental pollutants that accumulate and easily diffuse in soil and water systems [48,66,67]. The most common, perfluorooctancesulfonic acid, is accumulated by plants, and has been evaluated in numerous crop plants. As the examples summarized in Table 3 show, these chemical compounds demonstrate primarily localized effects on the tissues where they accumulate, though translocation to other plant organs is a common consequence of long-term exposure, as with HMs.

Some MPs present a more extreme example of a localized effect at the point of exposure than either HMs or the other types of chemical stress factors listed here. In a recent review, it was observed that the main effects of MP on plant growth are mechanical. MPs can adsorb to the surface of germinating seeds and roots, physically blocking water channels and nutrient uptake [50], respectively. The effects of MPs are not limited to the root system: they can enter into the xylem [51] where embolisms may form, further impeding water and nutrient translocation to the shoot tissues. Soil properties may also be altered by MP pollution, further reducing nutrient availability, soil respiration levels and pH [68]. By investigating the effect of polyethylene (PE)-MP of different diameters (13 µm and 130 µm) and different concentrations, it was found that alterations to soil properties were dependent on particle size. Smaller particles had a negative effect on soil respiration rates, dissolved oxygen content, and the availability of macronutrients phosphorus and potassium, and reduced soil pH. In contrast, larger particles still reduced potassium and phosphorus concentrations and pH, but soil respiration rates and dissolved organic carbon (DOC) were not impacted. Thus, changes to soil properties can have further effects on plant growth by limiting soil respiration and nutrient availability and overall nutrient cycling of the soil system.

### 4.2. From Localized to Systemic Stress: Salinity (Osmotic)

The amount of land area considered to be salt-affected (soils with greater than 40 mM sodium (Na) is rapidly increasing globally and becoming a widespread environmental concern (Table 1) [69]. Salinization is caused primarily by human activities and climate change and is now considered a critical driver of freshwater ecosystem degradation [70]. Saline soil conditions alter cell expansion and division, especially of the root system, limiting root growth [71]. Stomatal aperture is negatively affected by salinity-induced oxidative stress leading to their closure [54]. Elevated salinity significantly affects normal cellular ion homeostasis, specifically by decreasing K^+^/Na^+^ ratios [55,56]. Similar to drought, salinity stress also disrupts water relations and limits tissue hydration, and thus, activates similar response pathways [54,57]. Because of this, salinity stress is thought to impact plants in two different ways: (1) ion toxicity by accumulation of Na and limited uptake of K; (2) osmotic stress by disrupting tissue water potential. The response of plants to soil salinity can be localized to the root systems, in which the transport mechanisms that regulate Na uptake can aid plants in minimizing Na accumulation. The expression of some transporters, such as GmSOS1 in *Glycine max* [71] and HKT1:5 and NHX in *Oryza sativa* and *Oryza coarctata* [56] can be upregulated in response to exposure to elevated salinity to maintain optimal Na^+^ and K^+^ concentrations. Prolonged or intense exposure can severely disrupt Na^+^/K^+^ homeostasis, activating a systemic response that includes ROS signaling to initiate antioxidant production [72] and the hormone ABA to regulate stomatal aperture as a way to limit tissue dehydration [73].

As with HM stress, the dosage and the duration of the exposure to salinity is a key determining factor for the type of response that is elicited in the plant. Though the roots are the main point of direct contact with saline conditions, a more systemic response can be activated through stress signaling pathways, such as hormones and ROS, as Na^+^ is translocated to other tissues. Given their shared responses, metabolomic and genomic profiles could provide significant insight when HMs and salinity are applied individually and in combination.

### 4.3. Systemic Stresses with Hormonal Transduction Pathways: Drought, Flooding and Heat Stress

Abiotic stresses that are weather-related are among the most severe and widespread of environmental stresses, including drought [22], heat stress [74] and flooding [75,76]. All three are unpredictable in duration and their effects may be cumulative. Furthermore, climate change is increasing both their variability and intensity. For example, flooding can impact plants in different ways depending on the degree—waterlogging of the root system only, or partial or complete submergence of the shoot system. In flooded conditions, water fills the gas-filled pores in the soil, reducing soil oxygen levels leading to hypoxic conditions. The hypoxic conditions may be temporary with conditions returning to normal as water recedes [75,76]. As summarized in Table 3, reductions in plant growth are a general negative consequence of drought, flooding, and high temperatures. Limited root growth in all three scenarios further exacerbates the stress response by reducing nutrient uptake and availability to shoot-level processes such as photosynthesis and protein synthesis [59,60,63].

Direct exposure to stress is not necessary for plant tissues to exhibit a response. Though drought and flooding are mainly experienced at the root-level, responses are also seen in the shoots. In particular, hormones play an important role in communicating the imposition of stress to tissues that are not directly impacted. In many cases the transduction of these signals aids the plant in mounting defense mechanisms to mitigate the negative impact on the plant body overall. Abscisic acid (ABA) and ethylene are hormones whose roles in drought and flooding responses, respectively, are well known. ABA produced in the roots in response to reduced soil water potential values is transported to the shoots where, along with ROS and Ca signaling, it induces stomatal closure to prevent further water loss by plant [77]. Similarly, ethylene produced in submerged roots can stimulate the production of aerenchyma in non-submerged portions of the root system to increase oxygen availability for cellular respiration [76]. The inverse is true with heat stress (HS), in that though air temperature directly affects leaves and stems, the soil helps to buffer root systems from temperature extremes. Despite this, root system growth may also be negatively impacted under elevated air temperatures. Root growth is also significantly altered in response to high temperatures, with reductions in cell divisions in the root apical meristem and inhibition of lateral root growth [62]. In this example, both shoots and roots of *Arabidopsis thaliana* and *Solanum lycopersicon* (tomato) are negatively affected by elevated temperatures. Moreover, the study also showed that leaf temperature responses are influenced by the temperature that the roots experience in the soil. Using a specialized system to create realistic temperature gradients between the surface and the soil, it was found that HS signaling occurs between the root and shoot systems in *Arabidopsis thaliana* and tomato. When a temperature gradient was applied (cooler temperatures in the root zone than in the shoot zone), plants were better able to regulate their responses to maintain photosynthetic function at the leaf level. Based on the gene ontology data of the study, it appears that auxin signaling plays a role in communication of root temperature conditions to elicit a response at the shoot-level. For systemic stresses, plant hormones and ROS act as the messenger between the stress factor itself and the response, rather than having a subcellular effect based on distribution.

## 5. Combined Stress Responses: Shared and Unique Pathways

The plant response to combined stresses is complex, with specific symptoms and defense mechanisms differing depending on the specific combination to which the plant is exposed, the tolerance threshold of a particular species and the intensity of the stresses applied. In the following section we have characterized a set of studies from the literature regarding combined HM × abiotic stress responses in terms of whether the same degree of symptoms are induced and intensified (shared pathways—additive or synergistic, respectively) or whether the combination negates the symptoms of one at least one of the stresses or it elicits wider range of symptoms (unique pathways—antagonistic and novel stress responses, respectively) (Table 4).

From even this small sample of studies, a pattern begins to emerge that suggests correlations between the way the stress is applied to plant tissues (locally or systemically) and the type of response that is induced. For example, hydrocarbons, many of the MPs, and Na in glycophyte species may be absorbed and distributed throughout the plant via transporters. In the studies reviewed here, an additive or synergistic response is often observed with an exacerbation of the symptoms normally elicited by a single stress factor (HM, hydrocarbons, MPs and Na). In contrast, halophyte species, which possess mechanisms that aid in tolerating elevated soil salinity, generally show antagonistic responses to HM × salinity stress, often due to exclusion of the HM from the shoots and overall improved tolerance to both HM and Na. Drought, flooding and temperature stresses all rely on signaling pathways to elicit physiological responses in parts of the plant body not directly impacted by the stress. Many of these response pathways utilize plant hormones and ROS signaling to transmit information and to mount the production of defense compounds. Because of the variation among plants regarding the production and transport of hormones and other stress messengers, it is not surprising to see variation in the type of response that is initiated, ranging from additive to antagonistic. A survey of this nature highlights the necessity of taking HMs into consideration individually when evaluating combined HM × abiotic stress responses.

Further details of individual studies are provided in the subsections of Section 5, which are organized in terms of the main physiological process that is involved or affected by the combined stress.

### 5.1. Shared Pathways

#### 5.1.1. Tissue Damage and Reductions in Growth and Photosynthetic Activity

Among the most common responses to combined HM × abiotic stress are reductions in growth and other morphological alterations such as tissue chlorosis and necrosis. The combination of stresses may reduce the intensity of each stress that is needed to have a negative effect on these parameters than would occur if the stresses were applied individually. For example, the exposure of salt-sensitive strawberry (*Fragaria ananassa* Duch.) and lettuce (*Lactuca sativa* L.) to four salt concentrations (0–60 mM NaCl) and three Cd concentrations (0.3–5 mg Cd kg^−1^) showed a negative effect of salinity even at 20 mM NaCl, which induced leaf edge burn, chlorosis, necrosis and growth reduction. NaCl at 40 mM induced mortality in strawberry [86]. Tissue chlorosis is an indicator of chlorophyll pigment degradation and is a symptom induced under most HM combined stress conditions, especially in combination with MPs. A specific meta-analysis on Cd-MP interaction in soil–plant systems mainly showed that MPs have a negative effect on shoot and root biomass [83]. It was also observed that MPs promote Cd uptake and accumulation in plant shoots and roots, and that PE-MPs have a stronger effect on Cd accumulation than other types of MPs. This example demonstrates that the presence of MPs exacerbates the accumulation of Cd in the roots and its translocation to the shoots, reducing overall plant biomass.

A reduction in photosynthetic rate is commonly seen under individual and combined HM stress conditions. It may be the direct result of the induced degradation of chlorophyll pigments, reducing the capacity of photosynthetic tissues to absorb light energy to fuel CO_2_ fixation. However, several studies have highlighted instances in which other components of the photosynthetic process are directly impacted by the combination of HM with other abiotic stresses. Rubisco activity, the regulation of starch accumulation, and gas exchange capacity in terms of stomatal activity have all been found to be influenced by elevated HMs in combination with MPs. The impact of polystyrene (PS)-MP and Pb on mung bean (*Vigna radiata* L.) was studied, and it was found that in combination (40 μM Pb + 4.0 mg L^−1^ PS-MP), the stressors reduced the fresh and dry weight (DW) of shoots and roots, as well as Rubisco activity and chlorophyll content. The combined application of PS-MP and Pb also caused a decrease in important macromolecules, such as carbohydrates, lipids and proteins [80]. A pot experiment combined with computational chemistry explored the effects of polystyrene (PS)-MP and polytetrafluoroethylene (PTFE)-MP on As bioavailability in rice (*Oryza sativa* L.). The combined influence of PS-MP, PTFE-MP, and As caused an inhibition of soluble starch synthase and pyrophosphorylase activities in rice grains, resulting in a decrease in starch accumulation and biomass [81]. The single and joint effects of Cd and benzo(a)pyrene (B[a]P) on seedling growth and antioxidant enzyme activities of wheat (*Triticum aestivum* L.) were studied after 4, 8 and 11 days of exposure to combined stress of 10 mg L^−1^ Cd and 54 mg L^−1^ B[a]P. It was observed that after an 11-day exposure period, the combined stress induced reductions in Chl a, Chl b, Chl a + b and Chl a/b ratio. Net photosynthetic rate (Pn), transpiration rate (Tr), stomatal conductance (Gs), and intracellular CO_2_ concentration were considerably reduced as well [78]. The physical structure of chloroplasts may also be damaged, as found when two tomato genotypes were tested under conditions of control, waterlogging, Cd stress, and their combination [92]. It was found that the ultrastructure of tomato chloroplasts under individual and combined stress was damaged, and as a result, the plant height and biomass of the two genotypes decreased significantly.

#### 5.1.2. Increased Oxidative Stress Stimulates the Production of Protective Osmolytes and Antioxidants

The production of ROS is induced by heavy metals, drought, salinity, temperature, MP and flooding, making elevated ROS values a reliable marker of stress. Increased ROS concentrations in plant tissues can have wide ranging impacts that include lipid peroxidation, reduced membrane integrity and disruptions in signaling pathways. The damage due to ROS is often exacerbated by HM applied in combination with other abiotic stresses. In greenhouse-cultured rapeseed (*Brassica napus* L.), it was shown that MP (0.1%), combined with heavy metal treatments (50, 100 mg kg^−1^ Cu^2+^ or 25, 50 mg kg^−1^ Pb^2+^), significantly increased HM accumulation compared to HM treatments alone. Furthermore, severe damage and quality deterioration of rapeseed plants were caused by changes in MDA content, SOD and guaiacol peroxidase (GPX) activities, and sugar and vitamin C content [82].

To combat the overproduction of ROS induced by abiotic stress, the production of antioxidants is frequently upregulated, as well as that of other protective osmolytes like sugars and amino acids. When waterlogging and Cd stress were applied alone and in combination to two tomato genotypes, SOD activity increased under waterlogging and combined stress in genotype ‘MIX-002’, while under Cd stress in genotype ‘LA4440’ [92]. CAT activity decreased significantly in ‘MIX-002’ under waterlogging and in ‘LA4440’ under combined stress, while POD activity of ‘MIX-002’ increased under combined stress. The ascorbate peroxidase (APX) activity of the two genotypes was significantly lower under combined stress and higher than the respective controls, indicating that the plants were able to protect themselves from oxidative damage through the regulation of antioxidant enzymes in a genotype-dependent manner. A similar result was observed in two *Triticum aestivum* cultivars subjected to combined Cd and heat stress [93]. Total chlorophyll concentrations were reduced in both cultivars, but genotype-dependent differences in APX and CAT activity coincided with elevated expression of transcription factors that are involved in the regulation of abiotic stress responses and improved resistance to the combined stress in one of the cultivars. Antioxidant production and activity aid in the tolerance to abiotic stresses in general and appear to be highly variable in terms of the expression of related genes, making them a valuable selection marker.

Secondary metabolites, such as phenolic compounds, may also play an important defense role under HM combined stress conditions. Elevated temperatures in combination with heavy metals are expected to affect plant secondary metabolites; therefore, a study was conducted on *Robinia pseudoacacia* grown in Cd- and Pb-contaminated soil and exposed to elevated temperatures [94]. It was found that heavy metals increased the accumulation of saponins, phenolic compounds and flavonoids in leaves and stems, while elevated temperatures alone and in combination with Cd and Pb induced an overall increase in secondary metabolites, with phenolic compounds showing the greatest changes, suggesting that slightly elevated temperatures may enhance defense mechanisms in this species by stimulating the production of secondary metabolites.

#### 5.1.3. Exacerbation of Toxic Ion Uptake

Heavy metals, salinity and MPs can all be considered forms of chemical stress that have a toxic effect when accumulated in plant tissues. When combined with HM, elevated NaCl concentrations, MPs and hydrocarbons have been shown to increase plant stress symptoms by dramatically increasing the quantity of HM that are taken up by the plant, while simultaneously accumulating toxic concentrations of sodium, chloride and MPs. The overaccumulation of Na is a common salinity response in glycophytes, resulting in severe oxidative stress and reduced growth. This response is further exacerbated in the presence of various heavy metals. A study aimed at investigating the combined effect of salt and metal stress in *Vicia faba* L. plants by testing different concentrations of Pb and NaCl demonstrated that increased salinity causes an important accumulation of Na, particularly in the roots, and that high concentrations of NaCl and K in the culture medium induce potassium compartmentalization in the leaves [87]. Combined Cd and NaCl stress significantly increased Cd accumulation in the leaves of both strawberry (*Fragaria ananassa* Duch.) and lettuce (*Lactuca sativa* L.) [86]. Overall, there was a strong effect on ion uptake parameters with an overaccumulation of Na^+^ and Cl^−^ and a sharp decrease in K, Ca and Mg concentrations in different tissues. Higher translocation of Cd from root to shoot was found in lettuce compared to strawberry.

MP can adsorb heavy metals and increase phytotoxicity in plants; in fact, a general negative correlation has been found between MP concentration and size and heavy metal accumulation in plants, also observing that MP interact with heavy metals mainly by severely inhibiting plant growth, inducing oxidative stress damage, and affecting chlorophyll content and photosynthetic activity [95,96,97,98]. In greenhouse-cultured rapeseed (*Brassica napus* L.), it was shown that MP (0.1%), combined with heavy metal treatments (50, 100 mg kg^−1^ Cu^2+^ or 25, 50 mg kg^−1^ Pb^2+^), significantly increased HM accumulation compared to HM treatments alone. Furthermore, severe damage and quality deterioration of rapeseed plants were caused by changes in MDA content, SOD and GPX activities, and sugar and vitamin C content [82]. The action of different MPs, namely PE, polypropylene (PP), polylactic acid (PLA) and polybutylene adipate terephthalate, on soil microbial growth, Cd uptake and carbon metabolism has also been studied in *Brassica juncea* L., concluding that co-exposure to MPs and Cd stress can influence soil characteristics such as pH, microbial activity, Cd bioavailability, plant growth and Cd accumulation [98]. MPs negatively affect photosynthesis and increase oxidative damage, demonstrating that, in combination with Cd, they pose serious toxicity risks to plants.

### 5.2. Unique Pathways

The previous section highlighted instances where the combination of HM stress with a second abiotic has an additive, and in many cases, a synergistically negative effect on plant growth and other physiological processes. For some species an antagonistic response is observed in which the combination leads to a reduction in the effects normally induced by one or both of the abiotic stresses applied individually. The following section describes examples in which the application of combined stresses does not exacerbate the response and in some cases, counteracts the effects of one of the stresses applied.

#### 5.2.1. Biochemical Alleviation of Stress Response

Ionic and osmotic stresses like HM, salinity and drought are often observed to stimulate an increase in osmolytes that help to re-establish ion homeostasis and protect cellular machinery from ROS-induced oxidative stress [99]. These include amino acids like proline, sugars and glycine betaine. These compounds aid the plant in mitigating the stress response despite elevated accumulation of HMs or Na in root and shoot tissues. This is a phenomenon frequently observed among salt tolerant halophyte species. In *Atriplex canescens* seedlings grown in pots and subjected to NaCl (0.50 or 3%) and Pb(NO_3_)_2_ (800, 1600 or 2400 ppm), a high accumulation of Pb in the roots, a reduction in chlorophyll and an increase in proline in plant tissues were observed, but with a high general tolerance to these stress factors [88]. The halophytic shrub *Tamarix gallica* was grown for three months with an irrigation solution enriched with As (0, 200, 500 and 800 µM), with or without 200 mM NaCl [84]. As significantly reduced shoot growth; in contrast, pigment and nutrient contents (K^+^, Ca^2+^ and Mg^2+^) were not affected; therefore, *T. gallica* can be considered able to tolerate As intake (less than 500 µM) alone or in combination with NaCl.

#### 5.2.2. Exclusion Effect: Limited Uptake of HM

The mechanical blocking capacity of some microplastics can also have a positive effect when plants are simultaneously exposed to HMs. In some instances, the combination of HM and MP, including nanoparticles (NP), can reduce the adsorption of HMs, reducing tissue concentrations and preventing the oxidative stress that is normally the result of HM accumulation. With cucumber (*Cucumis sativus* L.) seedlings grown in Cr(VI)-contaminated soil, the effect of PE-, polyamide (PA)- and PLA-MP on Cr bioaccumulation and toxicity was investigated, observing a greater effect of MP on Cr accumulation in roots, stems and leaves than in fruits [100]. In fact, PE-MP increased, while PA-MP and PLA-MP reduced Cr accumulation in cucumber. In particular, high-dose and small-sized MP enhanced the effects of accumulated Cr on fresh weight and fruit yield.

#### 5.2.3. Induction of a Novel Effect

As many of the previous examples have shown, HM stress in combination with other abiotic stresses can result in an additive effect (Section 5.1), or in some instances, an antagonistic effect (Section 5.2) in which one stress mitigates the effect of the second. There are other cases in which novel effects are revealed only when multiple stresses are applied simultaneously, including the induction of unique patterns of stress hormone production or metabolic pathways [90]. Halophytes generally show improved growth even when HM uptake is not restricted under combined stress conditions. Two halophytes, *Atriplex halimus* and *Suaeda fruticosa*, were grown for one month with an irrigation solution enriched with 200 mM NaCl and 400 μM Cd^2+^ or 400 μM Cu^2+^ [101]. Biomass production and chlorophyll content were found to decrease under Cd stress in both species, while Cu showed a minor negative effect. The addition of NaCl to the irrigation solution improved plant performance, thus showing a mitigating effect on metal toxicity. It was also shown that both species accumulated significant amounts of Cd and Cu in the roots. Regarding the content of endogenous phytohormones, an increase in indole-3-acetic acid was observed in the roots of both species treated with high levels of Cd. The accumulation patterns of other phytohormones that aid in response to abiotic stress were more variable: salicylic acid (SA) levels in roots and leaves showed increases with high levels of Cd. This is in contrast to the reductions in both ABA and JA in the roots with elevated Cd, suggesting a specificity of certain hormones in response to HM stress.

In the literature, many examples can be found in which the combination of HM with MP or hydrocarbon toxicity leads to negatively synergistic effects on growth, with amplified uptake of the HM. A study on wheat documented that cadmium (40 mg L^−1^), alone or in combination with polypropylene (PP)-MP (50 and 100 μm), had a marginal effect on germination, while root and shoot lengths were reduced by Cd alone and in combination with PP-MP treatments. The antioxidant enzyme system of seeds and seedlings increased in the presence of the single Cd pollution, while the activities of SOD, CAT, and POD decreased in the presence of combined pollution [102]. A study on rice (*Oryza sativa* L.), grown in the presence of NP (0.001% *w*/*w*) and the metalloid arsenic (As) (1 mg L^−1^), alone or in combination, showed that NP had no effect on plant growth, but significantly increased As accumulation in shoots and roots by 70.9% and 24.5%, respectively. The toxicity caused by co-exposure to As and NP significantly reduced the ABA content in rice compared with As alone; furthermore, NP hindered the metabolism of SA, JA, and glutathione, thus enhancing oxidative phenomena and As translocation in the plant [85].

Other factors may modulate the intensity of the response to combined stress, such as the gender or age of the plant, or dosage level. Woody species are also subject to this type of stress. With the help of dendrochronology, the effect of environmental stressors on declining *Quercus robur* L. trees was studied. Through the analysis of temporal profiles of elements in earlywood tree rings, increased HM contamination was demonstrated under combined heavy metal and HS, with increasing levels of Pb and Cd and a lower resistance to environmental stressors of declining trees compared to healthy, growing trees. A negative relationship with temperature and evapotranspiration and a significant overall relationship between earlywood tree rings, climate and HM contamination were also highlighted [103]. The single and combined effects of Cd (5, 10 or 15 mg L^−1^) and fluoranthene (FLT) (1, 5 or 10 mg L^−1^) on germination, growth and photosynthesis of soybean (*Glycine max*) seedlings were studied. Exposure to these two compounds, individually or in combination, had a significant impact. It was shown that at low FLT concentrations, the interaction between Cd and FLT on germination was antagonistic, while the interaction was synergistic at higher FLT concentrations (5 or 10 mg L^−1^). Dry weight, root and shoot length, leaf area, net photosynthetic rate, intercellular CO_2_ concentration, chlorophyll content, and fluorescence of soybean seedlings were also reduced following treatment with 5 or 10 mg L^−1^ Cd or 1 or 5 mg L^−1^ FLT, individually or jointly, while both synergy and antagonism between Cd and FLT were observed for root growth [104].

The application of multiple stresses can, in some cases, stimulate a priming effect, reducing the effect of a second stress following the prior application of a different stress stimulus. It was observed that heat shock stimulated germination and growth in *Vigna mungo* seedlings, while Cd^2+^ treatment had an inhibitory effect [91]. It was also found that HS applied before cadmium treatment provided significant cross-protection to the Cd^2+^ effect up to 48 h. Regarding antioxidant defense enzymes, Cd^2+^ treatment moderately stimulated SOD activity, while HS slightly inhibited it.

## 6. Mitigation Strategies

**Figure 4:**
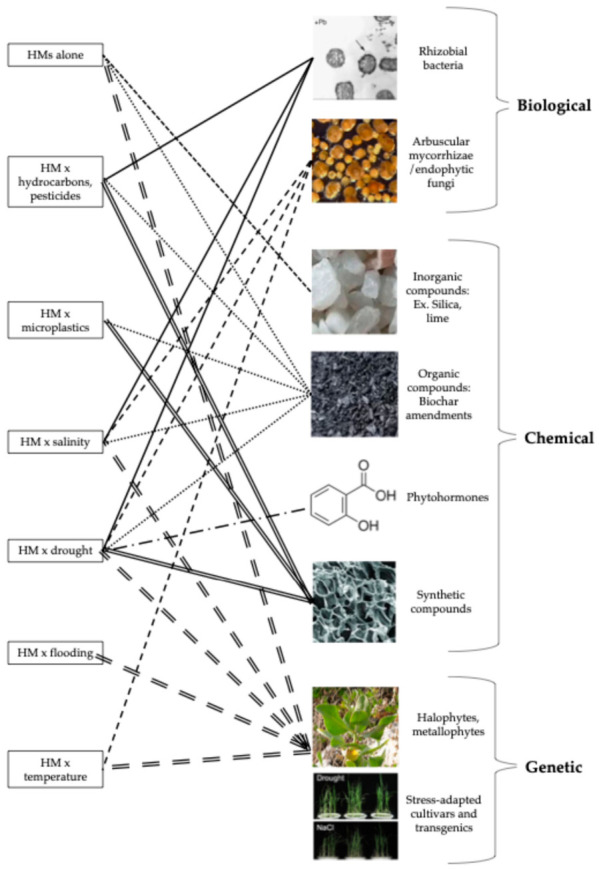
A summary of the mitigation strategies described in Section 6, linking strategies with their target stresses based on experimental evidence in the literature.

Mitigation strategies for various abiotic stressors can have a significant impact on the sustainability of agricultural and environmental systems. Integrated approaches that combine technological, biological, and genetic innovations to strengthen plant resilience to abiotic stress are always desirable and, among these, various materials have demonstrated interesting capabilities that should be appropriately exploited [105]. Some strategies are well-suited for a particular set of HM × abiotic stress combinations, while others represent a more general approach, as described in detail in the following section and summarized in Figure 4. For example, biochar, a natural soil amendment, has been found to be particularly effective at chelating HMs in the soil, reducing their uptake by plants. It is also effective at simultaneously reducing Na, hydrocarbon and MP pollution by stabilizing these compounds in the soil.

### 6.1. Biological

An intricate interplay exists between the microbiome and its host, particularly in the context of mitigating the adverse influences of climate change and abiotic stresses on plant resilience [106]. Root-borne bacteria and fungi are well-known to aid plants in acquiring water and nutrients when they are scarce and recent studies describe their role in improving plant growth in the presence of heavy metals as well. Several studies, in fact, describe the efficacy of plant microbiome in mitigating the effects of some abiotic stresses in plants. Among these are studies concerning heavy metals [107,108,109], drought [110,111], and salinity [112,113]. For example, a study on the interactions between nodule bacteria and *Pisum sativum* plants under drought and Cd stress found that rhizobial 1-aminocyclopropane-1-carboxylate (ACC) deaminase improved shoot biomass, nodulation, N_2_ fixation, and fertilizer (nitrogen and mineral nutrients) uptake in plants under single stress (water deficit or Cd toxicity), while combined water deficit and Cd stress negatively affected the plant/rhizobia interaction, which could not be improved by rhizobial ACC deaminase [114]. Endophytic fungi may also play a role in the plant response to combined HM × drought stress and HM x heat stress. The effect of *Paecilomyces formosus* LHL10 and *Penicillium funiculosum* LHL06 on *Glycine max* plants grown under heavy metal (Ni, Cd, and Al) stress, high temperature, and drought conditions was studied [115]. The authors found that co-inoculation with the fungi promoted plant growth and photosynthetic activity, as well as glutathione, catalase, and SOD activity, while reducing lipid peroxidation and improving macronutrient uptake under drought stress. Co-inoculation also reduced metal accumulation and translocation in plants by reducing heavy metal ATPase gene expression.

Arbuscular mycorrhizal fungi (AMF) are known to be key factors in plant–microbe interactions [116] and can help in promoting plant growth through various mechanisms, including improving water and nutrient absorption and mitigating the absorption of HM [117,118,119]. An interesting stress-alleviating effect of heavy metal-contaminated saline soils was attributed to the AMF *Funneliformis mosseae* (Fm) with the chelating agent nitrilotriacetic acid (NTA). A pot experiment on *Suaeda salsa* showed that AMF and NTA promoted plant growth, increased the accumulation of Na, Cd, and mineral elements, but decreased the concentrations of Na, Cd, and MDA in shoots when subjected to combined stress. Transcriptomic analysis revealed different regulatory pathways following single or combined application of the two amendments, identifying differentially expressed genes (DEGs) mainly in antioxidant defense, photosynthesis, and osmoregulation [120]. Alfalfa (*Medicago sativa*) fertilized with nitrogen or inoculated with a salt- and cadmium-tolerant *Sinorhizobium meliloti* strain was subjected to combined stress with NaCl and CdCl_2_, finding that inoculated plants had a higher aerial biomass than nitrogen-fertilized plants. SOD and CAT increased under stress conditions, but the increase in CAT activity was significantly lower in inoculated plants. Cd accumulation was also lower in inoculated plants. Expression analyses of the involved genes showed that inoculation increased resistance to combined stress by stimulating the biosynthesis of proline, phytochelatins and homophytochelatins [121].

Based on the knowledge that plant growth-promoting microorganisms can help improve plant resistance to stress conditions, research was conducted on the application of *Azospirillum* spp. (Az), phosphate-solubilizing bacteria (PSB), K-mobilizing bacteria (KMB), and AMF to rapeseed (*Brassica campestris*) plants subjected to salinity and Cd stress simultaneously. It was found that this combination of stresses primarily reduced plant growth, relative water content, and photosynthetic pigment levels, while increasing lipid peroxidation, electrolyte leakage, and ROS generation. In contrast, application of Az, PSB, or KMB restored these parameters to pre-stress values, with KMB being the most effective [122], underscoring the importance of K availability in confronting salinity stress.

General awareness of the dangers of micro- and nanoplastic bioaccumulation and hydrocarbon pollution in the environment is relatively recent. As such, to date there are few studies that have investigated approaches to mitigate the negative effects of MP, NP and hydrocarbon stress on plants alone or in combination with heavy metal stress. The complex interactions between heavy metals and PAHs must be taken into account, especially in relation to the activity of soil enzymes. In a pot experiment, alfalfa (*Medicago sativa* L.) plants were grown in soil containing moderate levels of HMs (Cu, Pb and Zn at 87, 100 and 110 mg kg^−1^ DW, respectively) and petroleum hydrocarbons (3800 mg kg^−1^ DW). The study evaluated the efficacy of alfalfa phytoremediation, *Pseudomonas aeruginosa* bioaugmentation and bioaugmentation-assisted phytoremediation. Analysis of plant biomass and physiological parameters demonstrated that plants were able to tolerate and grow in co-contaminated soil, especially when the soil was inoculated with *P. aeruginosa*, which appeared to reduce metal concentration and translocation into the plant and alleviate stress. The highest removal of petroleum hydrocarbons was obtained in the bioaugmentation-assisted phytoremediation treatment [123].

### 6.2. Chemical

The addition of stabilizing agents to contaminated soils reduces the bioavailability of HM through the formation of precipitates, HM complexes, and adsorption. This technique also offers other interesting advantages, such as increasing soil biological activity and its physicochemical properties, improving the soil’s water retention capacity, resulting in the proliferation and elongation of plant roots and thus greater availability of essential nutrients [124].

#### 6.2.1. Inorganic Compounds

Lime compounds are inorganic calcium-containing materials, characterized by a predominance of carbonates, oxides, and hydroxides. Lime stabilization treatment of hazardous waste-contaminated soils has yielded significant results and has become increasingly widespread over the years [125]. Silica, as well, exhibits versatile surface chemistry and high porosity with a large pore size, characteristics that give it good pollutant removal efficiency. More specifically, it functions effectively as an adsorbent for metal ion extraction due to its two-dimensional channel arrangement. The chemical and physical properties of silica-based adsorbents can also be improved through functionalization [126]. Other immobilizing agents, such as zeolite, clay minerals, iron oxides, and other chemical amendments [127,128] are frequently used in the case of HM toxicity in soils, but there is currently little information of their effectiveness under combined HM+abiotic stress conditions.

#### 6.2.2. Organic Amendments

Many studies have aimed to identify ways to mitigate the negative effects of combined heavy metal and other abiotic stresses, such as salinity and drought stress, through organic amendments. One of the most promising methods is the use of biochar (BC) as a soil amendment [129,130]. Biochar is the residual matter resulting from the burning of organic matter under low-oxygen conditions. It has gained significant attention recently in the last decade for its effectiveness in buffering the effects of both heavy metal and drought stress on plants and stabilizing heavy metals in the soil through chelation. The use of BC was studied in combination with zinc nanoparticles (ZnO NPs), finding that the application of ZnO NPs alongside co-composted BC was useful in reducing Cd uptake by *Triticum aestivum* plants under simultaneous Cd and drought stress [131]. Forty-five-day-old wheat plants were grown under conditions of severe and mild drought along with well-irrigated soil (control). The soil was enriched with three levels of BC (0%, 3.0% and 5.0% *w*/*w*). It was observed that drought stress increased Cd concentration in plants and caused oxidative stress associated with decreased antioxidant enzyme activities. It also negatively affected plant height, spike length, chlorophyll content, gas exchange parameters, root and shoot dry biomass and grain yield. In contrast, BC increased plant morphological and physiological parameters and reduced oxidative stress and Cd content. In combination with other natural and synthetic amendments, BC can improve plant growth despite simultaneous HM and drought stress [130]. The individual and combined effects of BC and plant growth-promoting rhizobacteria (PGPR) were studied on *Zea mays*. The authors observed improved growth and other physiology parameters (chlorophyll, phenolics, relative water content) and reduced accumulation of HM under drought, metal (Ni and Zn) or HM and drought conditions. It was concluded that the reduction in bioaccumulation of HMs from the combined treatment with groundnut shell biochar (GS-BC) and *Bacillus pseudomycoides* strain ARN7 suggests a possible use of the treatment to improve HM phytostabilization [89]. In a study on soil-grown wheat (*Triticum aestivum* L.) subjected to salinity and Cd accumulation, it was observed that, compared to Cd stress alone, soil salinity reduced several parameters such as plant growth, biomass, chlorophyll content, and gas exchange, and also caused oxidative stress [132].

Phosphorus-rich materials and HM chelators are widely used as soil remediation agents due to their high affinity for HMs present in the soil. Their use, alone or dispersed on porous BC (phosphorus-enriched biochar, PBC), has shown interesting applications in soil remediation. The preparation of engineered PBC composites, by incorporating components such as microorganisms, iron, double layered hydroxides, etc., also provides promising results [133]. Biodegradable chelators are interesting agents in the remediation of heavy metal. However, their use presents some challenges for their applications, such as the assessment of the factor affecting the efficiency of metal removal and the incomplete understanding of the involved mechanisms of metal removal [134]. Currently, the most often used and widely studied compounds are ethylenediaminetetraacetic acid [135], [S, S]–ethylenediaminedisuccinic acid [134], glucomonocarbonic acid [135], glutamate–N,N–diacetic acid [136], polyaspartic acid [137], and iminodisuccinic acid [138].

#### 6.2.3. Hormone Treatments

There are several natural and synthesized compounds that can be applied to reduce the negative effects of drought and heavy metal stress individually and in combination, helping plants to maintain photosynthetic pigments and avoid oxidative stress. The application of salicylic acid (SA), a growth regulator, and spermidine (Spd), a polyamine, can be used to counteract drought and chromium deficiency stress, either individually or in combination [139]. A study evaluated the efficacy of sodium nitroprusside (SNP) and melatonin (MEL) in alleviating the detrimental effects of drought and stress caused by excess Pb and Cd on maize growth. It was observed that under extreme water stress, MEL and SNP reduced nutrient uptake losses, especially potassium (K), while increasing biomass production, antioxidant activity and photosynthetic efficiency [140]. In date palm (*Phoenix dactylifera* L.), it was observed that exogenous silicon (Si) treatment in the root zone can reduce the stress caused by the combination of salinity and Cd toxicity, thus improving plant development and inducing physiological and biochemical modulation. In fact, Si application led to a lower metal uptake and an improvement in macronutrient uptake. MDA content was decreased through the modulation of antioxidant activities and endogenous stress-related hormones as ABA, SA and jasmonic acid (JA) were also downregulated [141].

#### 6.2.4. Synthetic Compounds

As several studies on MP have shown, synthetic compounds can actually have a mitigating effect with regard to HM stress by adsorbing and limiting their uptake. Other synthetic compounds can play a similar role, binding to soil contaminants or improving over plant growth in the face of HM and hydrocarbon pollution. A study on wheat [142] demonstrated that silicon (Si) nanoparticles applied through foliar dressing improved growth, photosynthesis, leaf defense system and Si concentrations in wheat tissues while reducing Cd content, especially in grains, in both normal and water-stressed soil containing excess Cd (7.67 mg kg^−1^). The lowest plant growth, yield and chlorophyll concentrations, associated with the highest oxidative stress and Cd concentrations in plant tissues were observed in the water-stressed control (35% WHC, water-holding capacity), followed by the normal control (75% WHC). A study focused on trying to limit the stress caused by Cd and DDT in *Brassica alboglabra* by testing the mitigating effect of Ca impregnated with benzenedicarboxylic acid (BdCa). BdCa indeed reduced the bioaccumulation of Cd and DDT, also supporting photosynthetic mechanisms and antioxidant enzymatic defenses [143]. Environmentally friendly compounds that aid plants in combatting the effects of abiotic stress are of increasing interest, especially compounds such as L-GH that may be synthesized from the by-products of industrial processes like papermaking. A pot experiment on Chinese broccoli (*Brassica campestris* L.), in fact, evaluated the effects of L-GH (up to 0.5%) and foliar Se (3 mg L^−1^) under drought conditions (H = 75% control; HA = 45% field water capacity) on growth and heavy metal (Cd, Cu, Zn) uptake. The results indicated that this treatment can effectively mitigate the combined stresses of drought and heavy metals on the growth of the analyzed plants [144]. Ryegrass was also tested in petroleum- and heavy metal-contaminated soil, with the application of nitrogen and phosphorus fertilizers [145]. The petroleum and heavy metal co-contamination promoted the increase in length, surface area, volume and diameter of ryegrass roots. Chl a/b and Chl/carotenoid ratios also increased, while the photosynthetic pigment content in the early stages of the experiment decreased. Fertilizer application improved the aboveground biomass growth, but reduced the length, surface area, volume and diameter of roots in the contaminated soil, concluding that fertilizers could alleviate the inhibitory effect of combined pollution, thus improving the remediation attitude of ryegrass.

### 6.3. Genetic

Different genetics approaches have been found to contribute to the mitigation of stresses due to the combined action of HM and other abiotic factors. Conventional breeding has relied on exploiting natural genetic variation for traits related to metal exclusion, sequestration, and tolerance, as well as for the entire complex array of phenomena associated with responses to other sources of abiotic stress. The effectiveness of this strategy, however, is often limited by the complex quantitative heritability and strong environmental influences on responses to these stresses. To overcome these limitations, modern breeding strategies integrate genomic selection, marker-assisted selection, QTL mapping, and the use of candidate genes to accelerate genetic progress and increase selection precision. Transgenic and genome-editing approaches are also expanding, allowing for the functional validation and targeted manipulation of numerous key genes primarily involved in antioxidant defense and stress signaling pathways [146]. Plant responses to abiotic stresses are clearly controlled by multiple traits and have typically been studied by analyzing single genes/traits. Genome-wide association studies (GWAS) now provide a more powerful tool for studying multiple or complex traits related to one or more stresses, providing useful information for breeding and developing future crops [147,148].

There are a number of targets for emerging genomic approaches to focus on in order to more fully understand the regulation of plant responses to abiotic stress, especially HMs in particular. Ion transporters play a key role in plant growth, development, and stress response. Based on this, several families of ion transporters from plants have been identified and characterized in recent years. These are being biotechnologically manipulated to better understand their functions. Furthermore, several studies aim to transfer or overexpress multiple genes to develop plants capable of withstanding multiple abiotic stresses [147,148]. MicroRNAs (miRNAs) and other RNAs are important post-transcriptional regulators modulating the expression of several stress-responsive genes [149]. Furthermore, plastid transformation is a useful transgenic approach for site-specific integration of foreign genes into the plastid genome and the stable expression of traits related to metal stress tolerance [150]. Among all available genome-editing principles aimed at creating novel quantitative trait loci for abiotic stress tolerance, the clustered regularly interspaced short palindromic repeat-Cas (CRISPR/Cas) system which can be utilized for rapid development of abiotic stress-tolerant plants and presents some positive aspects, such as simplicity, adaptability, flexibility and wide applicability [151].

Similar to the differences observed between glycophytes and halophytes, genotypic variation occurs in response to heavy metal and salinity stress, both individually and in combination. In two cotton (*Gossypium hirsutum* L.) genotypes differing in salt tolerance it was observed, in hydroponics, that salinity and/or Cd drastically reduced plant growth, chlorophyll content and photosynthesis with greater effect on the sensitive genotype. In this genotype salinity and/or Cd induced MDA accumulation, which instead remained unchanged in the tolerant genotype. In both genotypes, under combined stress Cd content was decreased while Na concentration was increased, compared with Cd alone, while differential responses of antioxidant enzymes to Cd, NaCl and combined treatments indicated genotype- and time course- dependent variations [152]. Transcription factors, in fact, are recognized for their significant involvement in both abiotic and biotic stress responses. These factors, combined with phytohormone signaling pathways regulated by compounds such as abscisic acid, salicylic acid, jasmonic acid, ethylene, and ROS, are important for effective cross-talk between different stress signaling pathways [153].

Transgenic *Arabidopsis* plants expressing the rice MYB-R1 transcription factor (OsMYB-R1) demonstrated increased tolerance to drought and chromium stress. The role of OsMYB-R1 in the expression of key stress-related genes, such as those that improve root architecture and in maintaining cellular homeostasis, has been highlighted [154]. In two rice cultivars (DR-92 and Bh-1, Cd tolerant and sensitive, respectively), the effect of increased Cd^2+^ levels and heat was studied, showing an increase in the activity of stress-related enzymes. In the presence of Cd^2+^ or heat stress individually, SOD and POD activities were higher in cv. DR-92, while CAT and glutathione reductase (GR) activities were higher in cv. Bh-1. In the presence of a combined Cd^2+^ and heat stress, SOD and POD activities decreased significantly in root/shoot in both sensitive and tolerant cultivars, while a strong increase in GR activity was detected in cv. Bh-1 [155]. In a study on transgenic rice (*Oryza sativa* L. cv. Zhonghua No.11) carrying glutathione-S-transferase (GST, EC.2.5.1.18) and catalase1 (CAT1, EC. 1.11.1.6), better resistance to Cd and combined Cd and heat stress was found in transgenics compared to non-transgenics, also demonstrating lower oxidative damage induced by Cd and combined stress in transgenics, measured not only by GST and CAT1 transgene but also by the whole ascorbate-glutathione cycle [156]. Even the introduction of the gene, BnbZIP3, from *Boehmeria nivea* into *Arabidopsis* helped to increase tolerance to cadmium and drought, as demonstrated by increased seed germination and root growth [157].

Transgenic tomato plants overexpressing SlMAPK3 showed significant stress tolerance due to the combination of drought and the presence of Cd (100 µM), highlighting a reduction in oxidative stress and an increased antioxidant defense [158,159]. The heavy metal ATPase (HMA), metallothionein (MT), and phytochelatin (PC) genes are essential for metal uptake, detoxification, and accumulation. Their combined contribution improves the efficiency of hyperaccumulation. Particularly MTs, cysteine-rich proteins with metal-chelating properties, have been shown to play a role in various abiotic stresses and are therefore designated as biomolecular markers of stress [160,161]. In chickpea overexpressing MTtype-1 (CarMT1) gene, the analysis of its role in stress tolerance against drought and heavy metals, it resulted that transgenic seeds showed a better seed survival efficiency under different heavy metal stresses and that high transcript levels were detected under the drought beyond changes in physiological and biochemical parameters, suggesting stress mitigating roles of CarMT1 [162].

It is also worth mentioning that several genetic mapping methods, such as quantitative trait loci (QTL) mapping, QTL sequencing (QTL-seq), and RNA sequencing (RNA-seq), have been developed to rapidly distinguish candidate genes within major QTLs. This work is being explored in the regulation of tolerance to various heavy metals [163].

## 7. Future Perspectives

The wide range of abiotic stresses to which plants can be subjected can significantly impact their survival. Heavy metal pollution, combined with other sources of stress, as illustrated, can have significant effects on various biological processes and severely alter normal plant growth. The effects can be synergistic, especially in case of salinity stress and HMs in glycophyte species and some MP and HM combinations across various species. Other HM and abiotic stress combinations can be antagonistic, with HM accumulation restricted, as in the case of some MPs, HS, and salinity in halophyte species. What emerges from the review of the studies included here is that the interaction between HMs and other abiotic stresses cannot be generalized into a single common response profile. Whether a response to a particular combined HM × abiotic stress combination is synergistic (exacerbated) or antagonistic (counteracted) likely depends on the way that individual metals are transported and accumulated in plant tissues, including translocation rates and sequestration mechanisms. In terms of stress combinations, there are links between the type of interaction the stress has with plant tissues (localized or systematic) and the response that is stimulated (synergistic or antagonistic). Combined localized stresses tend to elicit synergistic responses, while localized + systemic stresses are more variable, tending toward antagonistic. Further studies on the effects of combined stresses are needed, especially in relation to ongoing climate change and to address several aspects that may lead to variable results:

(1) the effect under field conditions: most studies of abiotic stresses are conducted under controlled conditions or in pot experiments. While this helps to isolate and characterize specific effects due to individual stresses, it is not necessarily the same response that would be seen when plants are exposed to multiple factors at once for varying periods of time and degrees of intensity. In nature, resources are rarely homogeneous in their availability, and plants exist within an ecosystem that includes soil, water, and air microorganisms that are all interacting and coping with the environmental conditions they are presented with;
(2) dosage of HM and other stresses can significantly shift the response from one that is synergistic to one that is additive, complicating the extent to which results from controlled trials can be interpreted;
(3) the potential role of priming in attenuating the effects of a later-imposed stress;
(4) the age of the plant when exposed to the stress can determine the outcome of the response. Some phases of the plant life cycle may be more sensitive to abiotic stresses than others, a factor that can have significant impacts on plant reproduction and survival, especially in an agricultural scenario when maximizing productivity is essential. It may also determine the appropriate timing to apply mitigation strategies in order to have the greatest impact on plant survival and productivity.

What is needed are approaches that will help us to improve our understanding of the effects of combined HM and abiotic stress responses in plants to find more targeted methods for improving crop plant adaptation (through classical breeding and biotechnology) and to apply the most efficient strategies for mitigating stress responses. The emerging use of multiomics approaches is an effective strategy, providing information about gene regulation networks, signaling pathways that are activated or deactivated, and the function of genes that are activated in response to a stress. For example, it appears from the studies on halophytes and metallophytes that an innate tolerance for one type of ionic stress tends to confer tolerance to a wider range of ion toxicity. One area of research that could provide a greater understanding of how this co-tolerance functions is through metabolomic analyses, a method that is already elucidating the intricate details of responses to individual stress. Molecular approaches have also been successfully applied under salinity and Cd stress conditions with the hydrophyte *Pistia stratiotes* [163], revealing significant changes in the metabolic profile and gene expression patterns. It was found that the addition of NaCl reduced Cd accumulation, and more importantly, transcriptome and weighted gene coexpression network (WGCNA) analysis revealed that the metabolic pathways of alpha-linolenic acid and biosynthesis of ribosomes, flavonoids, and phenylpropanoids were significantly enhanced under both treatments. Some genes were expressed only under salinity stress alone, while the combination of salinity and Cd stress led to an increase in the proteasome pathway.

Computer-based data integration and analysis are also valuable tools that may help to find patterns in responses from the vast amount of data that is already currently available. A compendium with an interactive platform, called “Stress Combinations and their Interactions in Plants Database” (SCIPDb; http://www.nipgr.ac.in/scipdb.php, accessed on 23 June 2026) has been conveniently created [164]. This platform provides information on morpho-physio-biochemical and molecular responses of plants to different stress combinations. Additionally, meta-analysis, CiteSpace and machine learning analyses have highlighted that co-exposure to MP/NP and HM can have contradictory toxic effects on plants, related to different factors [165].

## 8. Conclusions

In conclusion, plant responses to combined HM and abiotic stresses are complex, and in many cases contradictory, depending on the dosage and specific combination. There is a lot of information available from studies conducted under controlled conditions with experimental protocols that simplify the number of abiotic stress combinations. While this information is helpful in creating a general understanding of plant responses to multiple stresses, it does not provide a complete picture regarding the effects of these stresses under natural conditions. Climate change and the anticipated impacts to our food production system is a global problem. A global approach is needed to address the problem that should include sharing of the tools that are rapidly emerging to help integrate vast amounts of data, as well as new genetic technologies to help our food systems adapt and mitigation strategies to help combat the effects of these combined stresses in the field before reductions in yield have time to manifest. However, we can start now by applying the tools currently available in contexts that more closely resemble natural conditions to begin to fully elucidate the response networks that combined stresses elicit in plants.

## Figures and Tables

**Table 1:** A summary of the dominant abiotic stress factors that significantly impact the productivity of agricultural land. The amount of agricultural land that is impacted globally is indicated both in terms of the percent of total agricultural land and the number of hectares that this represents. For pesticides, herbicides and fungicides, there is a wide range of compounds used with varying degrees of impact on ecosystems. To simplify this category, only conventional and organic farming were considered.

	Subcategory (Where Applicable)	Amount of Agricultural Land		
Stress Factor		Percent of Total	Hectares	Reference
				
HM		14–17%	between 672,000,000–816,000,000	[2]
			
				
Salt	Irrigated	2.2%	105,600,000	[3]
	Rainfed	7.8%	374,000,000	
				
Drought	Moderate	30%	1,440,000,00	[4]
	Extreme	1.2%	57,600,000	
				
Flooding		10%	116,058,071	[5]
				
Temperature	Heat stress: >60 days above 35 °C during growing season	15.6%		[6]
			671,000,000	
	
			
	Cold stress: >30 days with minimum daily temp of 0 °C	7.3%		
			341,000,000	
	
				
Pesticides/ herbicides/ fungicides	Conventional farming	98.4%	4,700,000,000	[7]
	Organic farming	1.6%	77,000,000	[8]
			
				
Microplastics		No current estimates of land area		[9]
		1.5 to 6.6 million tonnes in soils globally		
			

**Table 2:** A summary of the topics and keywords used in the literature search.

**Search engine terms**			
	Themes	Keywords/Phrases	
	Heavy metals	arsenic, cadmium, cobalt, chromium, copper, nickel, lead	
	Abiotic stress	Drought, flooding, microplastics, salinity, synthetic chemicals (pesticides, fungicides, herbicides), temperature (heat, cold)	
	
	Types of responses	Additive, antagonistic, synergistic, positive, neutral, negative	
		
	Mitigation techniques	Biological mitigation methods, chemical mitigation methods, transgenics, biochar, synthetic compounds, phytohormones, endophytic fungi, arbuscular fungi, rhizobial bacteria	

	
**Criteria for study inclusion**			
	Themes	Inclusion	Exclusion
	Heavy metals	Individually As, Cd, Co, Cr, Cu, Ni, Pb	Al, Hg, Mn, Sr, Ti, Zn
		
		Any combinations of 1+ HMs that include at lease one of the above list	Any combinations without one of the included HMs, and those with 2+ of the excluded HMs (ex. Hg, Mn, Zn)
	
	
		
	Plants	Agricultural crop species/cultivars	Ornamental plant species
		
		Plants used as sustainable building materials	
		
		Crops of local importance	
		Edible plants with stress specific tolerance mechanisms	
		
	Type of trial	Greenhouse experiments, pot experiments	Hydroponic trials
		

**Table 3:** Principal plant responses to abiotic stresses, characterized according to the location of their main effect upon exposure.

Type of Stress	Environmental Factor	Specific Response [Ref]
Localized	Heavy metals	Summarized in Figure 1
	Hydrocarbons	Inhibition of macronutrient uptake and chlorophyll synthesis; oxidative stress [48]
	
	Synthetic chemicals	Decreased germination rates and plant growth; increased ROS production and membrane permeability [49]
	
		
Localized, mechanical	Microplastics	Reduced water and nutrient uptake of seeds and roots [50]; xylem embolism formation [51]; degradation of chlorophyll pigments, increased oxidative stress, disrupted protein function [52,53]

	
	
		
Localized → systemic	Salinity	ROS production, oxidative stress, stomatal closure [54]; disruption of Na/K homeostasis [55,56], tissue dehydration [57]

	
		
Systemic	Drought	Altered plant and water relations; reduced stomatal efficiency, rate of cell division and expansion, leaf size, stem elongation and root growth [54,57,58]
	
	
	Flooding	Reduced primary root growth and root system biomass [59,60]; reduced available oxygen shifts aerobic respiration to anaerobic; increased ethanol production; reduced protein synthesis [60]; root reductions lead to negative impacts to photosynthesis and chlorophyll content and oxidative stress [61]
	
	
	
	
	
	Heat	Reduced shoot and root growth [62]; disruption of carbon transport, storage and remobilization; altered sink capacity; reduced starch synthesis [63]; increased ROS [64]; meiotic cycle disruptions of gametes [65]
	
	
	

**Table 4:** A characterization of the primary effects of specific HM × abiotic stress combinations (additive, synergistic and antagonistic) with the principal mechanisms involved governing the response.

Stress Combination	Primary Effect	Mechanism Involved	Example		
			Metal	Species	Ref.
HM × hydrocarbons	synergistic	Signficant degradation of chlorophyll pigments; reductions in photosynthetic parameters	Cd	*Triticum aestivum* (L.)	[78]
		
				
	synergistic	Increase accumulation of Cd by pyrene	Cd	*Lolium perenne*	[79]
				
HM × microplastics	additive	Exacerbation of chlorophyll, protein, lipid and carbohydrate degradation	Pb	*Vigna radiata*	[80]
				
	additive	Reductions in starch storage and biomass	As	*Oryza sativa*	[81]
					
	synergistic	Significant induction of HM; oxidative stress	Cu; Pb	*Brassica napus*	[82]
				
	synergistic	Reductions in photosynthesis due to increased oxidative stress	Cd	multiple species	[83]
					
	synergistic	Increased uptake of Cr by PE-MP	Cr	*Cucumis sativus*	[84]
	antagonistic	Increased uptake of As; limited effect of NP	As	*Oryza sativa*	[85]
				
					
HM × salinity (glycophytes)	synergistic	Increased accumulation of Na and HM	Cd	*Fragaria ananassa*, *Lactuca sativa*	[86]
		
	synergistic	Increased Na accumulation with HM	Pb	*Vicia faba*	[87]
			Pb		
HM × salinity (halophytes)	antagonistic	HM accumulation restricted to roots; general tolerance to Na	Pb	*Atriplex canescens*	[88]
			
	antagonistic	Overall tolerance to both HM and Na	As	*Tamarix gallica*	[84]
HM × drought	synergistic	Similar growth to single stresses; significant reductions in chlorophyll, RWC and protein; significant increases in ROS scavenging mechanisms, membrane degradation	Ni	*Zea mays*	[89]
				
				
				
				
				
	antagonistic	Growth parameters under combined stress mirrored control or drought alone; photosynthetic parameters were similar to HM stress alone	Cu	*Lycopersicon lycopersicum*	[90]
HM × flooding	additive	Reduced biomass, chlorophyll degradation; oxidative stress	Cd	*Lycopersicon lycopersicum*	[82]
			
HM × temperature	antagonistic	HS stimulates cross-protection from Cd stress	Cd	*Vigna mungo*	[91]
			
	synergistic	Reduced chlorophyll pigments; increased ROS-scavenging antioxidants in cultivar with overexpression of TF for antioxidant enzymes	Cd	*Triticum aestivum* (L.) cultivars	[87]
			
				
				
				

## Data Availability

No new data were created or analyzed in this study. Data sharing is not applicable to this article.

## Abbreviations

The following abbreviations are used in this manuscript:

ABA	abscisic acid
ACC	1-aminocyclopropane-1-carboxylate
AMF	arbuscular mycorrhizal fungi
APX	ascorbate peroxidase
Az	*Azospirillum* sp.
B[a]P	benzo(a)pyrene
BC	biochar
BdCa	benzenedicarboxylic acid-impregnated Calcium
CAT	catalase
Chl	chlorophyll
DDT	dichlorodiphenyltrichloroethane
DEG	differentially expressed gene
DHAR	dehydroascorbate reductase
DOC	dissolved organic carbon
DW	dry weight
EC	electroconductivity
FLT	fluoranthene
GPX	guaiacol peroxidase
GR	glutathione reductase
Gs	stomatal conductance
GST	glutathione-S-transferase
GSTU	glutathione-S-transferase tau
GWAS	Genome-wide association studies
HM	heavy metal
HMA	heavy metal ATPase
HS	heat shock
JA	jasmonic acid
KMB	potassium-mobilizing bacteria
L-GH	lignin-based hydrogel
MDA	malondialdehyde
MTs	Metallothioneins
NTA	nitrilotriacetic acid
MEL	melatonin
MP	microplastics
NPs	nanoparticles
PA	polyamide
PAHs	polycyclic aromatic hydrocarbons
PBC	phosphorus-enriched biochar
PC	phytochelatin
PE	polyethylene
PEG	polyethylene glycol
PFAS	perfluoroalkyl and polyfluoroalkyl substances
PGPR	plant growth-promoting rhizobacteria
PLA	polylactic acid
Pn	net photosynthetic rate
POD	peroxidase
PP	polypropylene
PS	polystyrene
PSB	phosphate-solubilizing bacteria
PTFE	polytetrafluoroethylene
PVC	polyvinyl chloride
ROS	reactive oxygen species
RP	rubber particles
SA	salicylic acid
SNP	sodium nitroprusside
SOD	superoxide dismutase
Spd	spermidine
TOC	total organic carbon
Tr	transpiration rate
WHC	water-holding capacity

## References

[1] Bharti R., Sharma R. (2022). Effect of heavy metals: An overview. Mater. Today-Proc..

[2] Hou D., Jia X., Wang L., McGrath S.P., Zhu Y.-G., Hu Q., Zhao F.-J., Bank M.S., O’Connor D., Nriagu J. (2025). Global soil pollution by toxic metals threatens agriculture and human health. Science.

[3] FAO (2024). Global Status of Salt-Affected Soils—Main Report.

[4] Miao C., Zhang Q., Li X., Hu J., Sadegh M., AhgaKouchak A. (2026). Global drought extremes in 2025. Nat. Rev. Earth Environ..

[5] Zhang M., Zhai G., He T., Wu C. (2023). A growing global threat: Long-term trends show cropland exposure to flooding on the rise. Sci. Total Environ..

[6] Bajaj K., Mehrabi Z., Ramankutty N. (2025). Exposure of global agricultural lands to extreme weather using CMIP6 projections of future climate. Environ. Res. Lett..

[7] FAO (2005). Land Statistics 2001–2023—Global, Regional and Country Trends.

[8] Willer H., Trávníček J., Schlatter B. (2025). Statistical development of organic farming in global context. Advances in Organic Farming.

[9] Kedzierski M., Cirederf-Boulant D., Palazot M., Yvin M., Bruzaud S. (2023). Continents of plastics: An estimate of the stock of microplastics in agricultural soils. Sci. Total Environ..

[10] Macklin M.G., Thomas C.J., Mudbhatkal A., Brewer P.A., Hudson-Edwards K.A., Lewin J., Scussolini P., Eilander D., Lechner A., Owen J. (2023). Impacts of metal mining on river systems: A global assessment. Science.

[11] Nicholson F.A., Smith S.R., Alloway B.J., Carlton-Smith C., Chambers B.J. (2003). An inventory of heavy metals inputs to agricultural soils in England and Wales. Sci. Total Environ..

[12] Luo L., Ma Y., Zhang S., Wei D., Zhu Y.-G. (2009). An inventory of trace element inputs to agricultural soils in China. J. Environ. Manag..

[13] Adekanmi T.A. (2022). Health Hazards of Toxic and Essential Heavy Metals from the Poultry Waste on Human and Aquatic Organisms. Veterinary Medicine and Science.

[14] Munir N., Jahangeer M., Bouyahya A., El Omari N., Ghchime R., Balahbib A., Aboulaghras S., Mahmood Z., Akram M., Ali Shah S.M. (2022). Heavy metal contamination of natural foods is a serious health issue: A review. Sustainability.

[15] Rehman K., Fatima F., Waheed I., Akash M.S.H. (2018). Prevalence of exposure of heavy metals and their impact on health consequences. J. Cell Biochem..

[16] Jan A.T., Azam M., Siddiqui K., Ali A., Choi I., Haq Q.M. (2015). Heavy metals and human health: Mechanistic insight into toxicity and counter defense system of antioxidants. Int. J. Mol. Sci..

[17] Rai P.K., Lee S.S., Zhang M., Tsang Y.F., Kim K.H. (2019). Heavy metals in food crops: Health risks, fate, mechanisms, and management. Environ. Int..

[18] Mititelu M., Neacșu S.M., Busnatu Ș.S., Scafa-Udriște A., Andronic O., Lăcraru A.E., Ioniță-Mîndrican C.-B., Lupuliasa D., Negrei C., Olteanu G. (2025). Assessing heavy metal contamination in food: Implications for human health and environmental safety. Toxics.

[19] Rahman Z., Singh V.P. (2019). The relative impact of toxic heavy metals (THMs) (arsenic (As), cadmium (Cd), chromium (Cr)(VI), mercury (Hg), and lead (Pb)) on the total environment: An overview. Environ. Monit. Assess..

[20] Karri V., Kumar V., Ramos D., Oliveira E. (2018). Comparative in vitro toxicity evaluation of heavy metals (lead, cadmium, arsenic, and methylmercury) on HT-22 hippocampal cell line. Biol. Trace Elem. Res..

[21] Zandalinas S.I., Balfagón D., Gómez-Cadenas A., Mittler R. (2022). Plant responses to climate change: Metabolic changes under combined abiotic stresses. J. Exp. Bot..

[22] Hultgren A., Carleton T., Delgado M., Gergel D.R., Greenstone M., Houser T., Hsiang S., Jina A., Kopp R.E., Malevich S.B. (2025). Impacts of climate change on global agriculture accounting for adaptation. Nature.

[23] Liu F., Xi M., Liu T., Wu X., Ju L., Wang D. (2024). The central role of transcription factors in bridging biotic and abiotic stress responses for plants’ resilience. New Crops.

[24] Anwar K., Joshi R., Dhankher O.P., Singla-Pareek S.L., Pareek A. (2021). Elucidating the response of crop plants towards individual, combined and sequentially occurring abiotic stresses. Int. J. Mol. Sci..

[25] Shabbir R., Singhal R.K., Mishra U.N., Chauhan J., Javed T., Hussain S., Kumar S., Anuragi H., Lal D., Chen P. (2022). Combined abiotic stresses: Challenges and potential for crop improvement. Agronomy.

[26] Faridullah F., Pervaiz A., Irshad M., Alam A., Mahmood Q., Ashraf M. (2014). Trace elements characterization in fresh and composted livestock manures. J. Hydrol..

[27] Zhong X., Chen Z., Li Y., Ding K., Liu W., Liu Y., Yuan Y., Zhang M., Baker A.J.M., Yang W. (2020). Factors influencing heavy metal availability and risk assessment of soils at typical metal mines in Eastern China. J. Hazard. Mater..

[28] McCauley A., Jones C., Jacobsen J. (2009). Soil pH and organic matter. Nutrient Management Modules 8, #4449-8.

[29] Antoniadis V., Robinson J.S., Alloway B.J. (2008). Effects of short-term pH fluctuations on cadmium, nickel, lead, and zinc availability to ryegrass in a sewage sludge-amended eld. Chemosphere.

[30] Zeng F., Ali S., Zhang H., Ouyang Y., Qiu B., Wu F., Zhang G. (2011). The influence of pH and organic matter content in paddy soil on heavy metal availability and their uptake by rice plants. Ecotoxic Environ. Saf..

[31] Bourg A.C.M., Loch J.P.G., Salomons W., Stigliani W.M. (1995). Mobilization of heavy metals as affected by pH and redox conditions. Biogeodynamics of Pollutants in Soils and Sediments.

[32] Imseng M., Wiggenhauser M., Keller A., Müller M., Rehkämper M., Murphy K., Kreissig K., Frossard E., Wilcke W., Bigalke M. (2018). Fate of Cd in Agricultural Soils: A Stable Isotope Approach to Anthropogenic Impact, Soil Formation, and Soil-Plant Cycling. Environ. Sci. Technol..

[33] Marschner H. (2012). Mineral Nutrition of Higher Plants.

[34] Jin L., Qiao Q., Xiong Y., Liu Z., Wang Y., Guan E., Zhang P., Chen Z., Zhang L., Hou X. (2025). Absorption, transport, and bioavailability of lead (Pb) in plant systems and nanomaterial detoxification mechanisms. Plant Physiol. Biochem..

[35] Zhang X., Xue W., Zhang C., Wang C., Huang Y., Wang Y., Peng L., Liu Z. (2023). Cadmium pollution leads to selectivity loss of glutamate receptor channels for permeation of Ca^2+^/Mn^2+^/Fe^2+^/Zn^2+^ over Cd^2+^ in rice plant. J. Hazard. Mater..

[36] Mansoor S., Ali A., Kour N., Bornhorst J., AlHarbi K., Rinklebe J., Abd El Moneim D., Ahmad P., Chung Y.S. (2023). Heavy metal induced oxidative stress mitigation and ROS scavenging in plants. Plants.

[37] Yang S., Han X., Li J., Luan F., Zhang S., Han D., Yang M., Chen Q., Qi Z. (2024). *Oceanobacillus picturae* alleviates cadmium stress and promotes growth in soybean seedlings. J. Hazard. Mater..

[38] Rizvi A., Khan M.S. (2018). Heavy metal induced oxidative damage and root morphology alterations of maize (*Zea mays* L.) plants and stress mitigation by metal tolerant nitrogen fixing *Azotobacter chlorococcum*. Ecotoxicol. Environ. Saf..

[39] Wang L.-L., Ling F.-C., Qi Z.-A., Fang Z.-J., Li H., Gong J.-M. (2026). Localized nitrate uptake by NRT1.8 antagonizes root meristem growth inhibition upon cadmium stress. J. Hazard. Mater..

[40] Quadros I.P.S., Madeira N.N., Loriato V.A.P., Saia T.F.F., Silva J.C., Soares F.A.F., Carvalho J.R., Braga Reis P.A., Fontes E.P.B., Clarindo W.R. (2022). Cadmium-mediated toxicity in plant cells is associated with the DCD/NRP-mediated cell death response. Plant Cell Environ..

[41] Anjum N.A., Aref I.M., Duarte A.C., Pereira E., Ahmad I., Iqbal M. (2014). Glutathione and proline can coordinately make plants withstand the joint attack of metal (loid) and salinity stresses. Front. Plant Sci..

[42] Hamel L.-P., Sheen J., Séguin A. (2014). Ancient signals: Comparative genomics of green plant CDPKs. Trends Plant Sci..

[43] Jonak C., Nakagami H., Hirt H. (2004). Heavy metal stress. Activation of distinct mitogen-activated protein kinase pathways by copper and Cd. Plant Physiol..

[44] Fang H., Jing T., Liu Z., Zhang L., Jin Z., Pei Y. (2014). Hydrogen sulfide interacts with calcium signaling to enhance the chromium tolerance in *Setaria italica*. Cell Calcium.

[45] Khan T., Shah L., Khan S., Wani O.A., Sheikh Z.N., Afroza B., Rashid R., Baloch F.S., Mansoor S. (2025). Comprehensive review of multiomics applications and remediation of plant heavy metal toxicity. Stress. Biol..

[46] Vasilev A., Lidon F., Scotti P., Da Graca M., Yordanov I. (2004). Cd-induced changes in chloroplast lipids and photosystem activities in barley plants. Biol. Plant..

[47] Singh I., Shah K. (2014). Exogenous application of methyl jasmonate lowers the effect of Cd-induced oxidative injury in rice seedlings. Phytochemistry.

[48] Rico C.M., Wagner D.C., Ofoegbu P.C., Kirwa N.J., Clubb P., Coates K., Zenobio J.E., Adeleye A.S. (2024). Toxicity assessment of perfluorooctanesulfonic acid (PFOS) on a spontaneous plant, velvetleaf (*Abutilon theophrasti*), via metabolomics. Sci. Total Environ..

[49] Shahid M., Manoharadas S., Chakdar H., Alrefaei A.F., Albeshr M.F., Almutairi M.H. (2021). Biological toxicity assessment of carbamate pesticides using bacterial and plant bioassays: An in-vitro approach. Chemosphere.

[50] Jiang X., Chen H., Liao Y., Ye Z., Li M., Klobučar G. (2019). Ecotoxicity and genotoxicity of polystyrene microplastics on higher plant *Vicia faba*. Environ. Pollut..

[51] Li L., Luo Y., Li R., Zhou Q., Peijnenburg W.J.G.M., Yin N., Yang J., Tu C., Zhang Y. (2020). Effective uptake of submicrometre plastics by crop plants via a crack-entry mode. Nat. Sustain..

[52] Colzi I., Renna L., Bianchi E., Castellani M.B., Coppi A., Pignattelli S., Loppi S., Gonnelli C. (2022). Impact of microplastics on growth, photosynthesis and essential elements in *Cucurbita pepo* L.. J. Hazard. Mater..

[53] Shen L., Li Z., Huang X., Zhang P., Zhang L., Zhao W., Wen Y., Liu H. (2024). Effects of polystyrene microplastic composite with florfenicol on photosynthetic carbon assimilation of rice (*Oryza sativa* L.) seedlings: Light reactions, carbon reactions, and molecular metabolism. J. Hazard. Mater..

[54] Miller G., Suzuki N., Ciftci-Yilmaz S., Mittler R. (2010). Reactive oxygen species homeostasis and signaling during drought and salinity stresses. Plant Cell Environ..

[55] Ishikawa T., Shabala S. (2019). Control of xylem Na^+^ loading and transport to the shoot in rice and barley as a determinant of differential salinity stress tolerance. Physiol. Plant..

[56] Ishikawa T., Shabala L., Zhou M., Venkataraman G., Yu M., Sellamuthu G., Chen Z.-H., Shabala S. (2022). Comparative analysis of root Na+ relation under salinity between *Oryza sativa* and *Oryza coarctata*. Plants.

[57] Xu C., Shan J., Liu T., Wang Q., Ji Y., Zhang Y., Wang M., Xia N., Zhao L. (2023). CONSTANS-LIKE 1a positively regulates salt and drought tolerance in soybean. Plant Physiol..

[58] Farooq M., Wahid A., Kobayashi N., Fujita D., Basra S.M.A., Lichtfouse E., Navarrete M., Debaeke P., Souchère V., Alberola C. (2009). Plant drought stress: Effects, mechanisms and management. Sustainable Agriculture.

[59] Nguyen T.N., Tuan P.A., Mukherjee S., Son S., Ayele B.T. (2018). Hormonal regulation in adventitious roots and during their emergence under waterlogged conditions in wheat. J. Exp. Bot..

[60] Branco-Price C., Kaiser K.A., Jang C.J.H., Larive C.K., Bailey-Serres J. (2008). Selective mRNA translation coordinates energetic and metabolic adjustments to cellular oxygen deprivation and reoxygenation in *Arabidopsis thaliana*. Plant J..

[61] Chen K., Hu Q., Ma X., Zhang X., Qian R., Zheng J. (2024). The effect of exogenous melatonin on waterlogging stress in *Clematis*. Front. Plant Sci..

[62] González-García M.P., Conesa C.M., Lozano-Enguita A., Baca-González V., Simancas B., Navarro-Neila S., Sánchez-Bermúdez M., Salas-González I., Caro E., Castrillo G. (2023). Temperature changes in the root ecosystem affect plant functionality. Plant Commun..

[63] Hütsch B.W., Jahn D., Schubert S. (2019). Grain yield of wheat (*Triticum aestivum* L.) under long-term heat stress is sink-limited with stronger inhibition of kernel setting than grain filling. J. Agron. Crop Sci..

[64] Tian B., Talukder S.K., Fu J., Fritz A.K., Trick H.N. (2018). Expression of a rice soluble starch synthase gene in transgenic wheat improves the grain yield under heat stress conditions. Vitr. Cell. Dev. Biol.-Plant.

[65] Erickson A.N., Markhart A.H. (2002). Flower developmental stage and organ sensitivity of bell pepper (*Capsicum annuum* L.) to elevated temperature. Plant Cell Environ..

[66] Tariq S.R., Shafiq M., Chotana G.A. (2016). Distribution of heavy metals in the soils associated with the commonly used pesticides in cotton fields. Scientifica.

[67] Buck R.C., Franklin J., Berger U., Conder J.M., Cousins I.T., de Voogt P., Astrup Jensen A., Kannan K., Mabury S.A., van Leeuwen S.P.J. (2011). Perfluoroalkyl and polyfluoroalkyl substances in the environment: Terminology, classification, and origins. Integr. Environ. Assess. Manag..

[68] Cheng Y., Wang F., Huang W., Liu Y. (2024). Response of soil biochemical properties and ecosystem function to microplastics pollution. Sci. Rep..

[69] Jeppesen E., Beklioglu M., Ozkan K., Akyurek Z. (2020). Salinization increase due to climate change will have substantial negative effects on inland waters: A call for multifaceted research at the local and global scale. Innovation.

[70] Munns R., Tester M. (2008). Mechanisms of salinity tolerance. Ann. Rev. Plant Biol..

[71] Zhang M., Cao J., Zhang T., Xu L., Yang L., Li X., Ji F., Gao Y., Ali S., Zhang Q. (2022). A putative plasma membrane Na^+^/H^+^ antiporter GmSOS1 is critical for salt stress tolerance in *Glycine max*. Front. Plant Sci..

[72] Ketehouli T., Zhou Y.G., Dai S.Y., Carther K.F.I., Sun D.Q., Li Y., Nguyen Q.V.H., Hu X., Wang F.-W., Liu W.-C. (2021). A soybean calcineurin B-like protein-interacting protein kinase, *GmPKS4*, regulates plant responses to salt and alkali stresses. J. Plant Physiol..

[73] Chen K., Li G.J., Bressan R.A., Song C.P., Zhu J.K., Zhao Y. (2020). Abscisic acid dynamics, signaling, and functions in plants. J. Integr. Plant Biol..

[74] Zhao C., Liu B., Piao S., Wang X., Lobell D.B., Huang Y., Huanga M., Yaoa Y., Bassuk S., Ciais P. (2017). Temperature increase reduces global yields of major crops in four independent estimates. Proc. Natl. Acad. Sci. USA.

[75] Wright A.J., de Kroon H., Visser E.J.W., Buchmann T., Ebeling A., Eisenhauer N., Fischer C., Hildebrandt A., Ravenek J., Roscher C. (2017). Plants are less negatively affected by flooding when growing in species-rich plant communities. New Phytol..

[76] Pan J., Sharif R., Xu X., Chen X. (2021). Mechanisms of waterlogging tolerance in plants: Research progress and prospects. Front. Plant Sci..

[77] Liu H., Song S., Zhang H., Li Y., Niu L., Zhang J., Wang W. (2022). Signaling transduction of ABA, ROS, and Ca^2+^ in plant stomatal closure in response to drought. Int. J. Mol. Sci..

[78] Li Y., Chen Z., Xu S., Zhang L., Hou W., Yu N. (2015). Effect of combined pollution of Cd and B [a] P on photosynthesis and chlorophyll fluorescence characteristics of wheat. Pol. J. Environ. Stud..

[79] Li G., Wang Z., Lv Y., Jia S., Chen F., Liu Y., Huang L. (2021). Effect of culturing ryegrass (*Lolium perenne* L.) on Cd and pyrene removal and bacteria variations in co-contaminated soil. Environ. Technol. Innov..

[80] Chen F., Aqeel M., Khalid N., Nazir A., Irshad M.K., Akbar M.U., Alzuaibr F.M., Ma J., Noman A. (2023). Interactive effects of polystyrene microplastics and Pb on growth and phytochemicals in mung bean (*Vigna radiata* L.). J. Hazard. Mater..

[81] Dong Y., Bao Q., Gao M., Qiu W., Song Z. (2022). A novel mechanism study of microplastic and As co-contamination on indica rice (*Oryza sativa* L.). J. Hazard. Mater..

[82] Jia H., Wu D., Yu Y., Han S., Sun L., Li M. (2022). Impact of microplastics on bioaccumulation of heavy metals in rape (*Brassica napus* L.). Chemosphere.

[83] Huang F., Hu J., Chen L., Wang Z., Sun S., Zhang W., Jiang H., Luo Y., Wang L., Zeng Y. (2023). Microplastics may increase the environmental risks of Cd via promoting Cd uptake by plants: A meta-analysis. J. Hazard. Mater..

[84] Sghaier D.B., Duarte B., Bankaji I., Caçador I., Sleimi N. (2015). Growth, chlorophyll fluorescence and mineral nutrition in the halophyte *Tamarix gallica* cultivated in combined stress conditions: Arsenic and NaCl. J. Photoch. Photobio. Biol..

[85] Jiang Y., Chen X., Cao X., Wang C., Yue L., Li X., Wang Z. (2024). Mechanistic insight into the intensification of arsenic toxicity to rice (*Oryza sativa* L.) by nanoplastic: Phytohormone and glutathione metabolism modulation. J. Hazard. Mater..

[86] Ondrasek G., Rengel Z., Maurović N., Kondres N., Filipović V., Savić R., Blagojević B., Tanaskovik V., Gergichevich C.M., Romić D. (2021). Growth and element uptake by salt-sensitive crops under combined NaCl and Cd stresses. Plants.

[87] Azzouz F., Bouziani E. (2022). Impact of salt and metallic stress on the sodium and potassium uptake by the *Vicia faba* L. plants. Plant Arch..

[88] Ouaini A., Yssaad H.A., Nouri T., Nani A., Benouis S. (2023). Influence of combined stress by salinity (NaCl) and heavy metals (Pb (NO_3_)_2_) on the proline, chlorophyll and lead accumulation in the tissues of the *Atriplex canescens* (Pursh) Nutt. Agric. Sci. Technol..

[89] Anbuganesan V., Vishnupradeep R., Bruno L.B., Sharmila K., Freitas H., Rajkumar M. (2024). Combined application of biochar and plant growth-promoting rhizobacteria improves heavy metal and drought stress tolerance in *Zea mays*. Plants.

[90] Naz F., Hamayun M., Rauf M., Arif M., Afzal Khan S., Ud-Din J., Gul H., Hussain A., Iqbal A., Kim H.Y. (2022). Molecular mechanism of Cu metal and drought stress resistance triggered by *Porostereum spadiceum* AGH786 in *Solanum lycopersicum* L.. Front. Plant Sci..

[91] Kochhar S., Kochhar V.K. (2005). Expression of antioxidant enzymes and heat shock proteins in relation to combined stress of cadmium and heat in *Vigna mungo* seedlings. Plant Sci..

[92] Zhou R., Niu L., Yin J., Jiang F., Wang Y., Zhao T., Wu Z., Zhu W. (2023). Differences in physiological responses of two tomato genotypes to combined waterlogging and cadmium stresses. Antioxidants.

[93] Ergün N., Ozçubukçu S., Kolukirik M., Temizkan Ö. (2014). Effects of temperature—Hevy metal interactions, antioxidant enzyme activity and gene expression in wheat (*Triticum aestivum*) seedlings. Acta Biol. Hung..

[94] Zhao Y.H., Jia X., Wang W.K., Liu T., Huang S.P., Yang M.Y. (2016). Growth under elevated air temperature alters secondary metabolites in *Robinia pseudoacacia* L. seedlings in Cd-and Pb-contaminated soils. Sci. Total Environ..

[95] Kumar R., Ivy N., Bhattacharya S., Dey A., Sharma P. (2022). Coupled effects of microplastics and heavy metals on plants: Uptake, bioaccumulation, and environmental health perspectives. Sci. Total Environ..

[96] An Q., Wen C., Yan C. (2024). Meta-analysis reveals the combined effects of microplastics and heavy metal on plants. J. Hazard. Mater..

[97] Xu L., Xie W., Dai H., Wei S., Skuza L., Li J., Shi C., Zhang L. (2024). Effects of combined microplastics and heavy metals pollution on terrestrial plants and rhizosphere environment: A review. Chemosphere.

[98] Wang B., Wang P., Zhao S., Shi H., Zhu Y., Teng Y., Jiang G., Liu S. (2023). Combined effects of microplastics and cadmium on the soil-plant system: Phytotoxicity, Cd accumulation and microbial activity. Environ. Pollut..

[99] Sharma A., Shahzad B., Kumar V., Kohli S.K., Sidhu G.P.S., Bali A.S., Handa N., Kapoor D., Bhardwaj R., Zheng B. (2019). Phytohormones regulate accumulation of osmolytes under abiotic stress. Biomolecules.

[100] Gong K., Zhang Q., Shao X., Wu Y., Qiao Z., Qiu L., Zhang W., Peng C. (2024). Microplastics alter Cr accumulation and fruit quality in Cr (VI) contaminated soil-cucumber system during the lifecycle: Insight from rhizosphere bacteria and root metabolism. Sci. Total Environ..

[101] Bankaji I., Sleimi N., López-Climent M.F., Perez-Clemente R.M., Gomez-Cadenas A. (2014). Effects of combined abiotic stresses on growth, trace element accumulation, and phytohormone regulation in two halophytic species. J. Plant Growth Regulat..

[102] Han Z., Osman R., Liu Y., Wei Z., Wang L., Xu M. (2023). Analyzing the impacts of cadmium alone and in co-existence with polypropylene microplastics on wheat growth. Front. Plant Sci..

[103] Nechita C., Iordache A.M., Lemr K., Levanič T., Pluhacek T. (2021). Evidence of declining trees resilience under long term heavy metal stress combined with climate change heating. J. Clean. Prod..

[104] Li Q., Lu Y., Shi Y., Wang T., Ni K., Xu L., Liu S., Wang L., Xiong Q., Giesy J.P. (2013). Combined effects of cadmium and fluoranthene on germination, growth and photosynthesis of soybean seedlings. J. Environ. Sci..

[105] Rahim H.U., Ali W., Uddin M., Ahmad S., Khan K., Bibi H., Alatalo J.M. (2025). Abiotic stresses in soils, their effects on plants, and mitigation strategies: A literature review. Chem. Ecol..

[106] Kabir A.H., Baki M.Z.I., Ahmed B., Mostofa M.G. (2024). Current, faltering, and future strategies for advancing microbiome-assisted sustainable agriculture and environmental resilience. New Crops.

[107] Zheng Y.K., Qiao X.G., Miao C.P., Liu K., Chen Y.W., Xu L.H., Zhao L.X. (2016). Diversity, distribution and biotechnological potential of endophytic fungi. Ann. Microbiol..

[108] Pandey V.C. (2012). Phytoremediation of heavy metals from fly ash pond by *Azolla caroliniana*. Ecotoxicol. Environ. Saf..

[109] Hao L., Zhang Z., Hao B., Diao F., Zhang J., Bao Z., Guo W. (2021). Arbuscular mycorrhizal fungi alter microbiome structure of rhizosphere soil to enhance maize tolerance to La. Ecotoxicol. Environ. Saf..

[110] Zandalinas S.I., Mittler R., Balfagón D., Arbona V., Gómez-Cadenas A. (2018). Plant adaptations to the combination of drought and high temperatures. Physiol. Plant..

[111] Xu L., Naylor D., Dong Z., Simmons T., Pierroz G., Hixson K.K., Kim Y.-M., Zink E.M., Engbrecht K.M., Wang Y. (2018). Drought delays development of the sorghum root microbiome and enriches for monoderm bacteria. Proc. Natl. Acad. Sci. USA.

[112] Li X., Zhang X., Zhao Y., Yu X. (2020). Cross-talk between gamma-aminobutyric acid and calcium ion regulates lipid biosynthesis in *Monoraphidium* sp. QLY-1 in response to combined treatment of fulvic acid and salinity stress. Biores. Technol..

[113] He Q., Xie Z., Fu Z., Wang H., Chen L., Gao S., Zhang W., Song J., Xu P., Yu J. (2021). Effects of phenol on extracellular polymeric substances and microbial communities from aerobic granular sludge treating low strength and salinity wastewater. Sci. Total Environ..

[114] Belimov A.A., Zinovkina N.Y., Safronova V.I., Litvinsky V.A., Nosikov V.V., Zavalin A.A., Tikhonovich I.A. (2019). Rhizobial ACC deaminase contributes to efficient symbiosis with pea (*Pisum sativum* L.) under single and combined cadmium and water deficit stress. Environ. Exp. Bot..

[115] Bilal S., Shahzad R., Imran M., Jan R., Kim K.M., Lee I.-J. (2020). Synergistic association of endophytic fungi enhances *Glycine max* L. resilience to combined abiotic stresses: Heavy metals, high temperature and drought stress. Ind. Crops Prod..

[116] Shi X., Zhao Y., Xu M., Ma L., Adams J.M., Shi Y. (2024). Insights into plant–microbe interactions in the rhizosphere to promote sustainable agriculture in the new crops era. New Crops.

[117] Yang J., Kloepper J.W., Ryu C.M. (2009). Rhizosphere bacteria help plants tolerate abiotic stress. Trends Plant Sci..

[118] Chen L., Wang F., Zhang Z., Chao H., He H., Hu W., Fang L. (2023). Influences of arbuscular mycorrhizal fungi on crop growth and potentially toxic element accumulation in contaminated soils: A meta-analysis. Crit. Rev. Environ. Sci. Technol..

[119] Santander C., Aroca R., Ruiz-Lozano J.M., Olave J., Borie F., Cornejo P. (2017). Arbuscular mycorrhiza effects on plant performance under osmotic stress. Mycorrhiza.

[120] Cui X., Jia B., Diao F., Li X., Xu J., Zhang Z., Li F.Y., Guo W. (2022). Transcriptomic analysis reveals the molecular mechanisms of arbuscular mycorrhizal fungi and nitrilotriacetic acid on *Suaeda salsa* tolerance to combined stress of cadmium and salt. Process Saf. Environ..

[121] Pacheco-Insausti M.C., Ponce I.T., Quiñones M.A., Pedranzani H.E., Pueyo J.J. (2023). Effects of inoculation with stress-tolerant rhizobia on the response of alfalfa (*Medicago sativa* L.) to combined salinity and cadmium stress. Plants.

[122] Hasanuzzaman M., Alam M.M., Naz F., Rummana S., Siddika A., Sultana A., Sinthi F., Prasad P.V.V. (2024). Modulating reactive oxygen species and ion homeostasis for combined salt and cadmium stress tolerance in *Brassica campestris*: The role of beneficial microbes. Plant Stress.

[123] Agnello A.C., Bagard M., van Hullebusch E.D., Esposito G., Huguenot D. (2016). Comparative bioremediation of heavy metals and petroleum hydrocarbons co-contaminated soil by natural attenuation, phytoremediation, bioaugmentation and bioaugmentation-assisted phytoremediation. Sci. Total Environ..

[124] Tauqeer H.M., Fatima M., Rashid A., Shahbaz A.K., Ramzani P.M.A., Farhad M., Basharat Z., Turan V., Iqbal M., Hasanuzzaman M. (2021). The current scenario and prospects of immobilization remediation technique for the management of heavy metals contaminated soils. Approaches to the Remediation of Inorganic Pollutants.

[125] Derakhshan Nejad Z., Jung M.C., Kim K.H. (2018). Remediation of soils contaminated with heavy metals with an emphasis on immobilization technology. Environ. Geochem. Health.

[126] Cashin V.B., Eldridge D.S., Yu A., Zhao D. (2018). Surface functionalization and manipulation of mesoporous silica adsorbents for improved removal of pollutants: A review. Environ. Sci. Water Res. Technol..

[127] Lwin C.S., Seo B.H., Kim H.U., Owens G., Kim K.R. (2018). Application of soil amendments to contaminated soils for heavy metal immobilization and improved soil quality—A critical review. Soil. Sci. Plant Nutr..

[128] Peng W., Li X., Xiao S., Fan W. (2018). Review of remediation technologies for sediments contaminated by heavy metals. J. Soils Sediments.

[129] Burrows L.A., Edwards C.A. (2004). The use of integrated soil microcosms to assess the impact of carbendazim on soil ecosystems. Ecotoxicology.

[130] Abbas T., Rizwan M., Ali S., Adrees M., Mahmood A., Zia-ur-Rehmanet M., Ibrahim M., Arshad M., Qayyum M.F. (2018). Biochar application increased the growth and yield and reduced cadmium in drought stressed wheat grown in an aged contaminated soil. Ecotoxicol. Environ. Saf..

[131] Bashir A., ur Rehman M.Z., Hussaini K.M., Adrees M., Qayyum M.F., Sayal A.U., Rizwan M., Ali S., Alsahli A.A., Alyemeni M.N. (2021). Combined use of zinc nanoparticles and co-composted biochar enhanced wheat growth and decreased Cd concentration in grains under Cd and drought stress: A field study. Environ. Technol. Innov..

[132] Abbas T., Rizwan M., Ali S., Adrees M., Zia-ur-Rehman M., Qayyum M.F. (2018). Effect of biochar on alleviation of cadmium toxicity in wheat (*Triticum aestivum* L.) grown on Cd-contaminated saline soil. Environ. Sci. Pollut. Res..

[133] Peiris C., Alahakoon Y.A., Arachchi U.M., Mlsna T.E., Gunatilake S.R., Zhang X. (2023). Phosphorus-enriched biochar for the remediation of heavy metal contaminated soil. J. Agric. Food Res..

[134] Cao Y., Qian X., Zhang Y., Qu G., Xia T., Guo X., Jia H., Wang T. (2019). Decomplexation of EDTA-chelated copper and removal of copper ions by non-thermal plasma oxidation/alkaline precipitation. Chem. Eng. J..

[135] Beiyuan J., Tsang D.C., Valix M., Zhang W., Yang X., Ok Y.S., Li X.-D. (2017). Selective dissolution followed by EDDS washing of an e-waste contaminated soil: Extraction efficiency, fate of residual metals, and impact on soil environment. Chemosphere.

[136] Wang G., Zhang S., Xu X., Zhong Q., Zhang C., Jia Y., Li T., Deng O., Li Y. (2016). Heavy metal removal by GLDA washing: Optimization, redistribution, recycling, and changes in soil fertility. Sci. Total Environ..

[137] Mu’azu N.D., Haladu S.A., Jarrah N., Zubair M., Essa M.H., Ali S.A. (2018). Polyaspartate extraction of cadmium ions from contaminated soil: Evaluation and optimization using central composite design. J. Hazard. Mater..

[138] Tang J., Tang H., Wang H., Sima W., Liang R., Liao Y., Liang C. (2023). Enhanced electrokinetic remediation removal heavy metal from sludge assisted by the combined biodegradable iminodisuccinic acid and electrolyte circulation technology. Int. J. Environ. Anal. Chem..

[139] Naz R., Sarfraz A., Anwar Z., Yasmin H., Nosheen A., Keyani R., Roberts T.H. (2021). Combined ability of salicylic acid and spermidine to mitigate the individual and interactive effects of drought and chromium stress in maize (*Zea mays* L.). Plant Physiol. Biochem..

[140] Ullah F., Saqib S., Zaman W., Khan W., Zhao L., Khan A., Khan W., Xiong Y.-C. (2024). Mitigating drought and heavy metal stress in maize using melatonin and sodium nitroprusside. Plant Soil..

[141] Khan A., Bilal S., Khan A.L., Imran M., Al-Harrasi A., Al-Rawahi A., Lee I.J. (2020). Silicon-mediated alleviation of combined salinity and cadmium stress in date palm (*Phoenix dactylifera* L.) by regulating physio-hormonal alteration. Ecotox. Environ. Saf..

[142] Adrees M., Khan Z.S., Rehman M.Z., Rizwan M., Ali S. (2022). Foliar spray of silicon nanoparticles improved the growth and minimized cadmium (Cd) in wheat under combined Cd and water-limited stress. Environ. Sci. Pollut. Res..

[143] Mubeen S., Shahzadi I., Akram W., Saeed W., Yasin N.A., Ahmad A., Shah A.A., Siddiqui M.H., Alamri S. (2022). Calcium nanoparticles impregnated with benzenedicarboxylic acid: A new approach to alleviate combined stress of DDT and cadmium in *Brassica alboglabra* by modulating bioacummulation, antioxidative machinery and osmoregulators. Front. Plant Sci..

[144] Ma Q., Huang W., Xu W., Zhou H., Hashan D., She D. (2024). Synergistic mechanisms of lignin-based novel materials and leaf-surface selenium fertilizer in alleviating drought and heavy metal stress. Ind. Crop. Prod..

[145] Zhang C., Zhang Z., Zhou J., Wang Y., Ai Y., Li X., Zhang P., Zhou S. (2022). Responses of the root morphology and photosynthetic pigments of ryegrass to fertilizer application under combined petroleum–heavy metal stress. Environ. Sci. Pollut. Res..

[146] Hasan N., Naaz N., Budakoti M., Laskar R.A., Shariq M., Sharma N., Choudhary S., Bhinda M.S., Joshi D.C. (2026). Breeding approaches for enhancing heavy metal tolerance in legume: A comprehensive review. Plant Mol. Biol..

[147] He G., Qin L., Tian W., Meng L., He T., Zhao D. (2020). Heavy metal transporters-associated proteins in *Solanum tuberosum*: Genome-wide identification, comprehensive gene feature, evolution and expression analysis. Genes.

[148] Challa S., Neelapu N.R., Wani S.H. (2018). Genome-wide association studies (GWAS) for abiotic stress tolerance in plants. Biochemical, Physiological and Molecular Avenues for Combating Abiotic Stress Tolerance in Plants.

[149] Ghosh S., Adhikari S., Adhikari A., Hossain Z. (2022). Contribution of plant miRNAome studies towards understanding heavy metal stress responses: Current status and future perspectives. Environ. Exp. Bot..

[150] Koźmińska A., Wiszniewska A., Hanus-Fajerska E., Muszyńska E. (2018). Recent strategies of increasing metal tolerance and phytoremediation potential using genetic transformation of plants. Plant Biotech. Rep..

[151] Zafar S.A., Zaidi S.S.E.A., Gaba Y., Singla-Pareek S.L., Dhankher O.P., Li X., Mansoor S., Pareek A. (2020). Engineering abiotic stress tolerance via CRISPR/Cas-mediated genome editing. J. Exp. Bot..

[152] Ibrahim W., Ahmed I.M., Chen X., Cao F., Zhu S., Wu F. (2015). Genotypic differences in photosynthetic performance, antioxidant capacity, ultrastructure and nutrients in response to combined stress of salinity and Cd in cotton. Biometals.

[153] Li J., Tian J., Zhou M., Tian J., Liang C. (2024). Research progress on the physiological and molecular mechanisms underlying soybean aluminum resistance. New Crops.

[154] Tiwari P., Indoliya Y., Chauhan A.S., Pande V., Chakrabarty D. (2020). Over-expression of rice R1-type MYB transcription factor confers different abiotic stress tolerance in transgenic Arabidopsis. Ecotox. Environ. Saf..

[155] Nahakpam S., Shah K. (2011). Expression of key antioxidant enzymes under combined effect of heat and cadmium toxicity in growing rice seedlings. Plant Growth Regul..

[156] Zhao F.Y., Liu W., Zhang S.Y. (2009). Different responses of plant growth and antioxidant system to the combination of cadmium and heat stress in transgenic and non-transgenic rice. J. Integr. Plant Biol..

[157] Huang C., Zhou J., Jie Y., Xing H., Zhong Y., She W., Wei G., Yu W., Ma Y. (2016). A ramie (*Boehmeria nivea*) bZIP transcription factor BnbZIP3 positively regulates drought, salinity and heavy metal tolerance. Mol. Breed..

[158] Sreelatha L., Ambili A.L., Sreedevi S.C., Achuthavarier D. (2025). Metallothioneins: An unraveling insight into remediation strategies of plant defense mechanisms. Environ. Sci. Pollut. Res..

[159] Muhammad T., Zhang J., Ma Y., Li Y., Zhang F., Zhang Y., Liang Y. (2019). Overexpression of a mitogen-activated protein kinase SlMAPK3 positively regulates tomato tolerance to cadmium and drought stress. Molecules.

[160] Mierek-Adamska A.M., Tylman-Mojżeszek W., Pawełek A., Kulasek M., Dąbrowska G.B. (2025). The potential role of *Brassica napus* metallothioneins in salt stress and interactions with plant growth-promoting bacteria. Genes.

[161] Kumar S., Yadav A., Verma R., Dubey A.K., Narayan S., Pandey A., Sahu A., Srivastava S., Sanyal I. (2022). Metallothionein (MT1): A molecular stress marker in chickpea enhances drought and heavy metal stress adaptive efficacy in transgenic plants. Environ. Exp. Bot..

[162] Raza A., Tabassum J., Zahid Z., Charagh S., Bashir S., Barmukh R., Khan R.S.A., Barbosa F., Zhang C., Chen H. (2022). Advances in “omics” approaches for improving toxic metals/metalloids tolerance in plants. Front. Plant Sci..

[163] Wang S.Q., Zhou X.L., Jin Y.S., Jeppesen E., Yang L., Shen S.K. (2023). Gene co-expression networks unravel the molecular responses of freshwater hydrophytes to combined stress of salinity and cadmium. Chemosphere.

[164] Priya P., Patil M., Pandey P., Singh A., Babu V.S., Senthil-Kumar M. (2023). Stress combinations and their interactions in plants database: A one-stop resource on combined stress responses in plants. Plant J..

[165] Wu Y., Zhu J., Sun Y., Wang S., Wang J., Zhang X., Song J., Wang R., Chen C., Zou J. (2024). Effects of the co-exposure of microplastic/nanoplastic and heavy metal on plants: Using CiteSpace, meta-analysis, and machine learning. Ecotox. Environ. Saf..

